# Neutrophil Extracellular Traps in Viral Infections: Regulation, Immune Consequences, and Pathogenic Outcomes

**DOI:** 10.3390/cells15070580

**Published:** 2026-03-25

**Authors:** Clinton Njinju Asaba, Bella Nyemkuna Gwanyama, Humblenoble Stembridge Ayuk, Thomas Ikechukwu Odo, Razieh Bitazar, Tatiana Noumi, Patrick Labonté, Terence Ndonyi Bukong

**Affiliations:** 1 Armand-Frappier Santé Biotechnologie Research Center, Institut National de la Recherche Scientifique, Laval, QC H7V 1B7, Canada; clinton.asaba@inrs.ca (C.N.A.); thomas-ikechukwu.odo@inrs.ca (T.I.O.); razieh.bitazar@inrs.ca (R.B.); tatiana.noumi@inrs.ca (T.N.); patrick.labonte@inrs.ca (P.L.); 2 Department of Microbiology & Immunology, McGill University, Montreal, QC H3A 0G4, Canada; bella.gwanyama@mail.mcgill.ca; 3 Department of Environmental Immunology, Helmholtz Centre for Environmental Research-UFZ, 04318 Leipzig, Germany; humblenoble-stembridge.ayuk@ufz.de

**Keywords:** neutrophils, NETosis, viral infection, innate immunity, SARS-CoV-2, HBV, immunothrombosis, hyperinflammation, fibrosis, autoimmunity

## Abstract

Neutrophils are among the early responders of the innate immune system and play a key role in host defense against viral infections. Beyond their classical antimicrobial functions, neutrophils can engage in a specialized defense mechanism by releasing web-like extracellular DNA known as neutrophil extracellular traps (NETs). These extracellular traps are a mesh-like network of chromatin DNA decorated with cellular components, including histones, proteases, and antimicrobial enzymes, that function to contain and limit the spread of pathogens. While NET formation contributes to antiviral immunity, accumulating evidence indicates that excessive or dysregulated NET formation can significantly contribute to immunopathology during viral infections. Thus, depending on the context and outcome, NET formation may be viewed as a double-edged sword. Therefore, understanding the regulatory mechanisms governing NET formation and its harmful effects is critical for developing therapeutic strategies that enhance antiviral defense while minimizing tissue damage. In this review, we provide a comprehensive overview of the molecular mechanisms that drive NET formation and clearance, with a particular focus on how viruses modulate these processes to influence disease outcome. We also discuss the pathways underlying NET formation and subsequent neutrophil cell death (NETosis), including canonical and non-canonical pathways, and highlight key signaling axes involving SYK, MAPKs, and NF-κB. Using SARS-CoV-2 and hepatitis B virus as representative models, we examine how different viral components trigger, exploit, or evade NET targeting and how persistent accumulation of NETs can contribute to hyperinflammation, progressive tissue injury, and post-viral syndromes. We further explore emerging evidence linking impaired NET clearance and neutrophil heterogeneity, particularly low-density neutrophils (LDNs), to chronic inflammation and post-viral sequelae such as long COVID and autoimmune hepatitis. Finally, we summarize current and emerging therapeutic strategies aimed at modulating NET formation or enhancing NET clearance. Altogether, this review underscores the dual nature of NETs in viral infections, highlighting their potential roles in antiviral defense and tissue injury, and provides a framework for the development of targeted interventions to limit virus-induced immunopathology.

## 1. Introduction

Neutrophils are the most abundant innate immune cells in human circulation and serve as a critical first line of defense against invading pathogens [1]. This process involves the production of web-like structures called neutrophil extracellular traps (NETs), composed of decondensed chromatin that is decorated with histone proteins, antimicrobial peptides, and granular enzymes such as myeloperoxidase (MPO) and neutrophil elastase (NE) [1]. NET formation can trap and neutralize pathogens that are particularly large or evasive to conventional phagocytosis. Traditionally recognized for their roles in phagocytosis and degranulation, neutrophils also deploy a unique antimicrobial mechanism, known as NETosis, that results in their death. While these NETs are crucial for host protection and prevention of pathogen spread, their dysregulated formation or impaired clearance have been increasingly implicated in the pathogenesis of a wide range of inflammatory and autoimmune disorders [1,2,3].

Since the discovery of NETs by Brinkmann et al. in 2004 [4], numerous studies have provided evidence of their antimicrobial functions, while others have explored the key mechanisms regulating their formation [5]. Beyond their established role in defending against bacterial pathogens, recent research indicates that viral infections can also significantly influence NET production, particularly infections caused by respiratory and hepatotropic viruses such as severe acute respiratory syndrome coronavirus 2 (SARS-CoV-2) [6] and hepatitis B virus (HBV) [7], respectively.

The mechanisms by which viruses trigger or evade NET targeting are complex, involving direct viral-neutrophil interactions that engage pattern recognition receptors (PRRs) [6,8]. This engagement initiates downstream signaling cascades that lead to the generation of reactive oxygen species (ROS) [6], mobilization of intracellular calcium, and activation of mitogen-activated protein kinases (MAPKs) [9]. In numerous instances, viral proteins or nucleic acids act as potent triggers of NET formation [8,9], thereby amplifying the inflammatory responses that underlie severe viral pathogenesis. In the context of respiratory viral infections such as SARS-CoV-2 [8], influenza A [10], and respiratory syncytial virus (RSV) [11], excessive NET formation has been linked to tissue injury, vascular occlusion, and hyperinflammation. Moreover, multiple clinical studies have reported elevated levels of circulating NET components such as cell-free DNA (eDNA), MPO-DNA complexes, and citrullinated histone H3 in the plasma of severe COVID-19 patients [12,13,14]. In addition, single-cell RNA sequencing (scRNA-seq) analyses of bronchoalveolar lavage (BAL) fluid from COVID-19 patients have revealed upregulated expression of genes associated with NET formation, particularly in severe cases [15]. These observations underscore the pathogenic role of NETs, which can compromise alveolar and endothelial integrity, promote microthrombosis, and amplify cytokine production, thereby establishing a self-perpetuating cycle of inflammation and tissue injury.

In hepatotropic viral infections, such as those caused by HBV and HCV, neutrophils and NETs play dual roles in antiviral defense and liver injury [7,16,17]. A recent study has elucidated mechanisms by which HBV may modulate NET formation to promote hepatocellular carcinoma (HCC) [7]. The authors reported elevated NET levels in HCC patients with high HBV-DNA loads compared with those with lower viral loads [7]. There, in vitro and ex vivo experiments demonstrated that HBV induces the expression of the calcium-binding protein S100A9 in HCC cell lines (HepG2 and HepG2.2.15), which acts as a potent neutrophil activator, triggering NET formation. The study further demonstrated that HCC cells stimulate NET generation, which in turn promotes HCC progression by enhancing tumor cell proliferation, metastasis, and angiogenesis [7]. While NETs may help restrict viral replication, their persistent activation in the liver microenvironment can cause hepatocellular damage, increase fibrosis, and progression toward chronic liver inflammation [7,16]. Despite being among the first responders during infection, elevated neutrophil counts have been associated with poor prognostic outcomes in several viral infections, including SARS-CoV-2 [15] and hepatitis viruses [7]. Notably, the presence of high levels of neutrophil chemo-attractants/activators such as CXCL8, S100A8, and S100A9 in severe COVID-19 cases [15] and HBV-positive HCC patients [7] may contribute to excessive neutrophil recruitment and NET formation, potentially exacerbating disease severity and tissue injury. Moreover, impaired clearance of NETs by macrophages or hepatocytes can lead to sustained exposure to self-immunogenic nuclear antigens, triggering autoimmune reactions resembling those observed in long COVID or autoimmune hepatitis (AIH) [18,19,20]. In COVID-19, elevated levels of neutrophils exhibiting an immature or low-density (LDN) phenotype have been reported, particularly in severe cases [15,21,22]. These dysfunctional LDNs differ from conventional high-density neutrophils (HDNs) in their ability to secrete higher levels of proinflammatory cytokines (IL-1β, TNF-α) and type I or type II interferons, showing reduced phagocytic activity, enhanced ROS production, and spontaneous release of NETs, while remaining viable, thus escaping efficient clearance by macrophages (efferocytosis) [23,24]. Similarly, in patients with HBV-related liver failure, neutrophils exhibited impaired phagocytosis, increased degranulation, and augmented NET formation. These features predispose patients to inflammation-driven tissue injury and heightened susceptibility to secondary bacterial infections [25,26]. Therefore, it is plausible to hypothesize that severe COVID-19 or chronic HBV infection may give rise to a subset of phenotypically and functionally altered neutrophils resembling LDNs, which could contribute to persistent inflammation, immune dysregulation, and ineffective viral clearance.

NET formation is often accompanied by neutrophil cell death (NETosis), necessitating rapid clearance by scavenger cells such as macrophages through a process known as efferocytosis [27]. This step is essential for preventing collateral tissue injury caused by cytotoxic NET components and for maintaining tissue homeostasis. Emerging evidence suggests that defective efferocytosis or inefficient NET clearance can lead to the accumulation of toxic NET-derived molecules, including MPO, NE, and citrullinated histone, which may persist in tissues or the circulation, contributing to endothelial damage, inflammation, and organ dysfunction [24,28,29]. These components, such as histones, proteases, and oxidized DNA fragments, can act as damage-associated molecular patterns (DAMPs), amplifying inflammation through receptor-mediated pathways involving toll-like receptors (TLRs) or the cGAS-STING axis [29,30,31]. Therefore, the failure to efficiently remove NET remnants represents a key pathogenic mechanism linking excessive NET formation to sustained inflammation, endothelial dysfunction, thrombosis, and autoimmune sequelae.

While DNase-mediated degradation of NET chromatin supports macrophage efferocytosis and resolution, viruses can undermine this protective axis by exploiting NET-derived molecules for attachment and entry [27,32]. The resulting inflammation may further delay NET clearance, enabling continued exposure to NET components and sustaining a permissive environment for viral spread [32]. The pathological consequences of dysregulated NET formation and impaired clearance can extend beyond the acute phase of infection. Persistent or excessive NET activity can drive chronic inflammation, pulmonary fibrosis, impair optimal respiratory function, and the development of autoimmune diseases such as rheumatoid arthritis and systemic lupus erythematosus, as observed in some long COVID patients (a condition characterized by the persistence or emergence of new symptoms at least three months after the initial infection and lasting for a minimum of two months) [2,18]. In the liver, prolonged exposure to NET components can activate hepatic stellate cells, stimulate collagen deposition, and promote fibrotic remodeling, ultimately leading to cirrhosis or hepatocellular carcinoma [26,33,34,35]. Altogether, these findings underscore the dual nature of NETs as both defenders against infection and potential mediators of chronic tissue injury and autoimmunity when inadequately regulated.

Taken together, understanding how viruses modulate NET formation and clearance would provide crucial insights into the fine balance between protective immunity and pathogenic inflammation. The dual nature of NETs, as both defenders and destroyers, places them at the center of viral immunopathogenesis. Therefore, elucidating the molecular mechanisms that drive these processes during viral infections will not only deepen our understanding of host–pathogen interactions but also open avenues for targeted therapeutic strategies. Interventions that modulate NET formation or enhance their clearance remain promising for mitigating virus-induced inflammation, preventing tissue damage, and reducing the risk of autoimmune complications.

Literature Search Strategy: Using the NCBI PubMed database, we searched for articles published between 2008 and 2026 using terms focused on neutrophil biology and neutrophil extracellular traps (NETs), including neutrophils, NETs/NETosis, virus–NET interactions, SARS-CoV-2– and hepatitis B virus (HBV)–associated neutrophil phenotypes, neutrophil post-viral sequelae (including Long COVID), neutrophil–macrophage crosstalk, efferocytosis, alcohol-related liver disease and NETs, NET signaling regulation and bystander effects, and NET-targeted therapies and clinical trials. Retrieved records were screened and selected for relevance to NETs, NETosis, and efferocytosis in viral infections, with a primary emphasis on SARS-CoV-2 and HBV. Recognizing that NETs also contribute to shared pathomechanisms across multiple viral diseases, we additionally summarized relevant findings from other viral infections in a dedicated table to support a coherent translational synthesis and to provide a concise cross-virus comparison aligned with the review’s clinical and preclinical scope.

## 2. Viral Induction and Modulation of Neutrophil Extracellular Trap (NET) Formation

Upon viral infection, a localized innate immune response is rapidly initiated to recruit effector cells for viral clearance. In SARS-CoV-2 infection, this process is primarily orchestrated by lung epithelial cells and alveolar pneumocytes, which constitute the first line of defense in the respiratory tract [36]. Following viral entry, successful infection and replication, these cells release a variety of inflammatory cytokines (IL-1β, IL-6, TNF-α, IL-18, G-CSF) [37,38] and chemokines (CXCL1, CXCL2, CXCL5, CXCL8, CXCL10, CCL2, CCL3, CCL4) [36,37] that collectively mediate the recruitment and activation of diverse immune cell populations, including monocytes, macrophages, and neutrophils. Among these mediators, CXCL8 (IL-8) is a key chemokine that recruits and activates neutrophils. In settings of sustained inflammation, often driven by a high viral load or persistent infection, ongoing neutrophil influx and priming can increase the likelihood of NET formation [39,40].

Recent studies have shown that severe COVID-19 is associated with elevated CXCL8 expression by multiple immune cell subsets, including monocytes and macrophages, correlating with the high neutrophil-to-lymphocyte ratios observed in critically ill patients [15,40]. Similarly, during HBV infection, IL-15 and IL-7 produced by hepatic stellate cells and hepatocytes can enhance T cell activation and license them to secrete CXCL8, thereby indirectly amplifying neutrophil recruitment and activation within the liver microenvironment [41]. This cytokine-driven amplification loop may contribute to excessive NET formation, NETosis, and tissue injury, highlighting a common pathogenic axis linking respiratory and hepatic viral infections to dysregulated neutrophil function.

**Figure 1:**
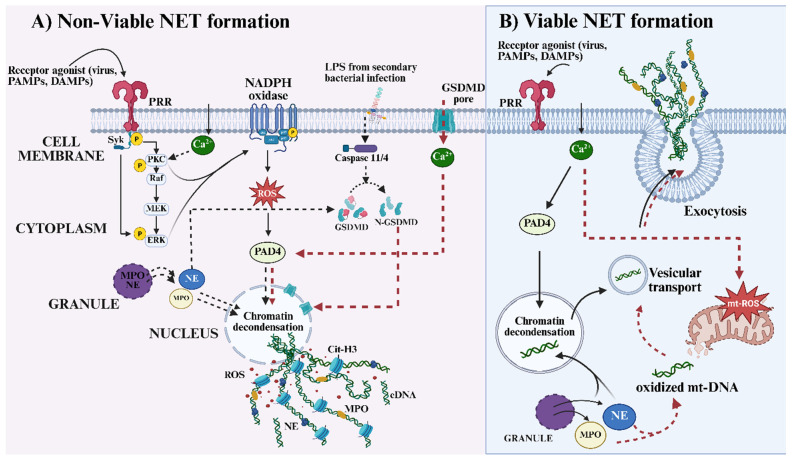
Mechanisms of non-viable and viable NET formation. (**A**) **Non-viable NET Formation**: Canonical, ROS-dependent NETosis (solid black arrows) is initiated by PRR (TLRs, CTLRs) engagement, leading to Ca^2+^ mobilization and activation of SYK and other downstream kinases (PKC-RAF-MEK-ERK) involved in NADPH oxidase activation/assembly. The phosphorylation of the p47 cytosolic subunit of NADPH oxidase by PKC or ERK promotes its activation and assembly, leading to ROS generation. ROS and/or Ca^2+^ activate PAD4, which mediates chromatin decondensation through histone citrullination (Cit-H3). ROS drives the granular release of NE and MPO, with NE further contributing to chromatin relaxation through histone proteolysis. The coordinated processes result in nuclear envelope breakdown and the lytic release of decondensed chromatin decorated with granule protein, forming the molecular structure of NET. In parallel, a non-canonical, ROS-independent pathway (red broken arrows) can be triggered by LPS from secondary bacterial infection, activating caspase-11 (in mice) or caspase-4 (in humans). Caspase-mediated cleavage of gasdermin D promotes membrane pore formation, Ca^2+^ influx, and ionic imbalance, thereby facilitating PAD4 activation, granule destabilization, and chromatin decondensation. Beyond caspase activity in the noncanonical pathway, NE has also been shown to cleave gasdermin D. Both canonical and non-canonical pathways ultimately converge on lytic NET release followed by NETosis. (**B**) **Viable NET Formation:** In contrast to non-viable NET formation, vital (non-lytic) NET formation preserves plasma membrane integrity and neutrophil viability. Vital NETosis (solid black arrows) is triggered by receptor engagement and Ca^2+^ influx, leading to PAD4-dependent chromatin decondensation without nuclear envelope breakdown. Nuclear DNA and granular proteins are packaged into vesicles and released extracellularly as NETs. A second vital pathway involves mitochondrial NET formation (red broken arrows), in which Ca^2+^ influx and further ionic signaling induce mitochondrial stress, generation of mitochondrial ROS (mtROS), and mitochondrial membrane permeabilization. This results in the release of oxidized mitochondrial DNA (mtDNA), which binds granule proteins with affinity. mtDNA and granule proteins are packaged together and exported via vesicular mechanisms. This pathway proceeds independently of NADPH oxidase activity, histone citrullination, and nuclear disruption, yielding NETs composed predominantly of mtDNA. Created in Biorender. Gwanyama, B. (2026) https://BioRender.com/52h3aqu.

Neutrophils express a broad repertoire of PRRs, including surface and endosomal Toll-like receptors (TLRs) as well as cytosolic RNA or DNA sensors [42,43], which collectively detect structural and genetic components of viral particles and initiate downstream immune activation. Viruses, just like other pathogens and damaged cellular molecules, possess distinct molecular signatures that function as pathogen-associated molecular patterns (PAMPs) or DAMPs, enabling their recognition by innate immune sensors on neutrophils (see Figure 1). The engagement of one or more receptors on neutrophils by viral components can activate a network of downstream signaling pathways that ultimately culminate in NET formation [9,43,44]. Reports have shown that TLR2 and TLR4, expressed on the neutrophil surface, recognize viral envelope proteins and glycoproteins such as the SARS-CoV-2 spike and RSV fusion proteins, triggering signaling cascades involving MyD88, SYK, MAPKs, and NF-κB [9,44,45]. In addition, other pattern recognition receptors, particularly C-type lectin receptors such as CLEC5A and Dectins, have been shown to serve similar roles in viral sensing and neutrophil activation [45]. These converging pathways drive the transcription of proinflammatory mediators (IL-1β, TNFα, IL8) and promote the activation of the NADPH oxidase complex, leading to the generation of ROS [6,44], a critical prerequisite for ROS-dependent NET formation (see Figure 1). Beyond surface receptors, neutrophils also detect viral components through endosomal and cytosolic sensors [6]. Endosomal TLR7/8 recognize viral ssRNA [6], whereas TLR9 detects unmethylated CpG DNA [43,46]. In the cytoplasm, RIG-I-like receptors (RIG-I, MDA5) sense viral RNA species and signal through MAVS and IRF3/7, promoting interferon production and amplifying antiviral responses [6,47]. The resulting cytokine milieu, particularly elevated levels of interleukin-8 (IL-8/CXCL8), tumor necrosis factor-alpha (TNF-α), and type I/II interferons, further recruits and activates neutrophils, thereby increasing their tendency to undergo NETosis during viral infections [6,43,48].

Research on the immunobiology of neutrophils has highlighted their ability to mediate antibody-dependent cellular cytotoxicity (ADCC), a mechanism through which Fc receptors recognize and bind to the Fc portion of antibodies coating target cells or pathogens, resulting in their elimination through cytotoxic processes [49]. However, during certain viral infections, this pathway can be aberrantly activated, leading to the formation of immune complexes composed of viral antigens bound to antibodies [50,51]. These immune complexes engage Fcγ receptors, particularly FcγRIIA (CD32) and FcγRIIIB (CD16B), expressed on neutrophils, triggering SYK- and PKC-dependent signaling cascades [52]. The downstream activation of these pathways promotes chromatin decondensation, nuclear envelope rupture, and the subsequent release of NETs [51,52], thereby linking Fc receptor engagement and immune complex formation to NETosis in the context of viral infection.

In the case of SARS-CoV-2, opsonized viral particles and immune complexes have been shown to potentiate NET formation through the non-receptor tyrosine kinase (SYK) signaling pathway [52]. Although the exact signaling mechanisms by which the virus or its components initiate NET formation remain elusive, other studies have identified possible downstream targets of activated SYK [53]. Once activated, SYK can phosphorylate multiple downstream molecules, including phospholipase C gamma (PLCγ), which catalyzes the generation of diacylglycerol (DAG) and inositol trisphosphate (IP3) [53]. This leads to activation of protein kinase C (PKC) and intracellular calcium mobilization, which are essential for NADPH oxidase activation and subsequent ROS production [54]. The resulting oxidative burst promotes peptidyl arginine deiminase type IV (PAD4)-mediated histone citrullination and chromatin decondensation, culminating in the extracellular release of NETs through the classical ROS-dependent pathway [54] (see Figure 1). On the other hand, NET formation can also be induced via ROS-independent mechanisms. In this case, SYK-PKC signaling can activate alternative pathways involving calcium influx-driven PAD4 activation [55], Lamin B disassembly [56], or engagement of caspase and gasdermin D-dependent signaling [57], leading to chromatin decondensation and nuclear envelope rupture independent of NADPH oxidase activity.

Depending on the type of virus or the nature of the infection, and on the surrounding cytokine milieu, neutrophils may initiate either ROS-dependent or ROS-independent pathways to induce NET formation. Therefore, it would be crucial to identify the molecular factors that influence these pathways in different viruses to uncover the mechanisms underlying virus-specific modulation of neutrophil responses. In addition to shedding light on how to mitigate, exploit, or evade neutrophil responses, this knowledge would guide the development of targeted therapies to mitigate abnormal/excessive NET formation and its associated inflammatory and autoimmune sequelae.

### Viral Evasion and Exploitation of Neutrophil Extracellular Traps

Although NETs can provide beneficial antiviral defense mechanisms that help protect the host from virus damage, some viruses have evolved strategies to evade NET-mediated targeting or hijack NET components to ensure their own survival, infectivity, and persistence [32,58]. These evasion strategies may involve either blocking NET formation or promoting the degradation of NET structures and their molecular components.

A common immune evasion strategy employed by some hepatotropic viruses, such as HBV, involves functionally inhibiting critical signaling molecules that drive NET formation. For example, studies have shown that chronically HBV-infected mice exhibited a reduced capacity for neutrophil-mediated bacterial clearance [58]. Moreover, key HBV structural proteins, including the core (HBc) and envelope (HBe) proteins, have been shown to suppress the phosphorylation and activation of essential signaling pathways such as ERK1/2, p38 MAPK, and mTOR, which are pivotal for ROS-dependent NET formation in healthy primary neutrophils [58]. Therefore, by targeting these molecular cascades, HBV can effectively dampen NET formation, contributing to an immunosuppressive environment that supports viral persistence and secondary infections. Despite these insights, the specific receptors and upstream recognition mechanisms through which HBV proteins interact with neutrophils remain poorly understood. Elucidating these receptor-ligand interactions will be crucial for uncovering how HBV or other viruses modulate neutrophil functions. Such new knowledge can be further exploited in developing therapeutic strategies aimed at restoring effective innate immune responses during viral infection.

Beyond directly inhibiting signaling molecules involved in NET formation, certain viruses, such as SARS-CoV-2, have evolved the ability to exploit NET components to facilitate their own infection. Recent reports show that the full-length histone H3 and H4, released during NET formation, can form multivalent interactions with both the S2 domain of the SARS-CoV-2 spike protein and sialic acid residues on host cell surfaces [32]. This dual binding mechanism effectively bridges the virus to host cells, thereby enhancing viral attachment and infectivity. While this emerging evidence implicates NETs in promoting SARS-CoV-2 infection, it is plausible that the marked neutrophilia observed in severe COVID-19 [32] may, at least in part, result from the virus’s capacity to induce various chemotactic and proinflammatory factors that drive excessive neutrophil recruitment and activation. Consequently, activated neutrophils can mount robust NET formation, which the virus may hijack to facilitate its spread and persistence, further aggravating tissue damage and inflammatory responses within the lung microenvironment.

Collectively, these evasion mechanisms highlight the evolutionary arms race between host antiviral defenses and viral countermeasures. While NET formation can provide an effective mechanism to limit viral spread, viral suppression of NET formation or hijacking NET components can favor chronic infection, enhanced infectivity, and immune evasion. Understanding this balance is crucial for designing targeted therapies that modulate NETs activity, increasing protective roles while limiting tissue injury during viral infections.

## 3. Consequences of Dysregulated NET Formation and Persistent NET Components During Viral Infection

Most NET-associated materials originate from the nucleus of neutrophils, which contain substantial amounts of nuclear, cytosolic, and granular proteins. As previously mentioned, NET formation involves the release of various intracellular components, such as MPO, NE, proteinases, histones, and calcium-binding S100 proteins [17,41,59]. These molecules are embedded into the reticular structure of NETs, forming the basis of their physiological and pathological functions. While these components play essential roles in antiviral defense and the maintenance of tissue homeostasis, their excessive release due to dysregulated NET formation can have deleterious effects on surrounding tissues [21,26,35]. Moreover, depending on the disease context or pathology, neutrophils can adopt distinct pathogenic phenotypes and functional states [21,22,25,60]. Specifically, neutrophils may assume a phenotype that drives a broad spectrum of pathological outcomes, including chronic inflammation, tissue damage, immunothrombosis, autoimmunity, and even multi-organ failure [2,3,18,24,35]. It has been reported that neutrophil involvement in immunopathology following viral infection is driven by their impaired clearance [15,59]. Emerging evidence suggests that this may be due to neutrophil reprogramming, which is largely driven by distinct transcriptional changes and the type of viral infection [61,62]. Most notably, several studies have shown that the appearance of neutrophils with immature (particularly LDNs) or senescent phenotypes during SARS-CoV-2 [15,21,63] or HBV [25] infection can contribute to increased inflammation through spontaneous NET production, heightened inflammatory cytokine release, and reduced clearance by macrophages. The exposure and persistence of these NET components to surrounding tissues have also been described as potential drivers of chronic immune activation and long-term tissue remodeling, particularly in the context of post-acute sequelae of viral infections [7,26,61], including post-acute sequelae of COVID-19 (PASC or Long COVID) [3]. Therefore, sustained NET accumulation, resulting from both excessive formation and defective clearance, may represent a central mechanism linking acute viral immune dysregulation to prolonged inflammatory pathology. This persistent NET burden could amplify local and systemic inflammation, promote endothelial dysfunction, and maintain a feed-forward loop of neutrophil activation, ultimately contributing to the chronic symptomatology observed in affected individuals. Considering these clinical observations, it is essential to deepen understanding of the molecular mechanisms by which NET-derived components mediate tissue injury and systemic pathology during and after viral infections, thereby guiding the development of targeted therapies.

Although SARS-CoV-2 [15,21,63] and HBV [7,25] are used here as representative models, virus-induced NET formation and impaired NET clearance have been reported in different viral infections. Other respiratory viruses, such as the influenza A virus [10,64] and RSV [11,65] have been reported to induce robust NET formation that contributes to severe lung injury. Similarly, reports indicate that arboviruses, including dengue [66] and chikungunya [67] viruses can induce NET-dependent vascular and inflammatory pathology. In addition, picornaviruses and retroviruses such as CVB3 [68] and HIV-1 [69] have also been shown to modulate NET formation and clearance, contributing to chronic inflammation and organ dysfunction (see Table 1). These observations indicate that NET-mediated immunopathology may represent a conserved feature of viral infections rather than a virus-specific phenomenon.

### Mechanistic Insights into NET-Induced Systemic and Organ Dysfunction

Although NET formation is a critical component of antimicrobial defense, it can also contribute to systemic inflammation and multi-organ dysfunction if not properly regulated. In this section, we outline and discuss the mechanisms by which excessive or persistent NET-derived molecules act as DAMPs, thereby propagating tissue injury and initiating diverse local and systemic pathological conditions.

**Figure 2:**
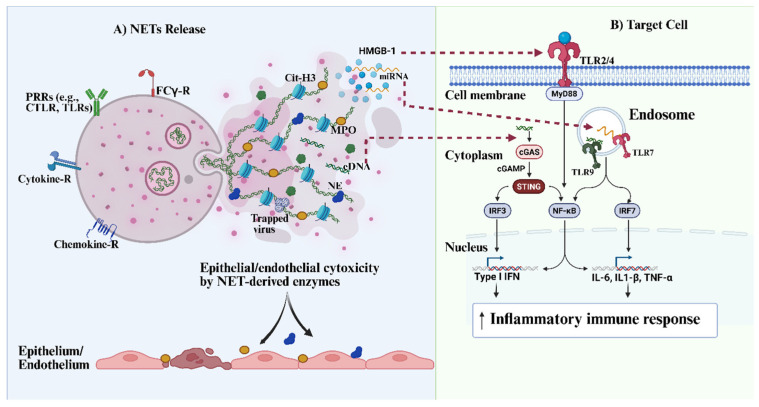
Schematic representation illustrating how excessive/persistent NET components contribute to local tissue injury and systemic immune dysregulation during viral infection. (**A**) **NET release and local cytotoxicity:** Upon activation through different PRRs, FC-γ receptors, cytokine and chemokine receptors, neutrophils undergo NETosis, releasing a meshwork of extracellular matrix composed of nuclear or mitochondrial DNA decorated with granular and cytosolic proteins. The externalized NETs comprise various molecules, including eDNA, MPO, NE, Cit-H3, HMGB1, and microRNAs. While NETs can entrap and limit viral dissemination, excessive NET formation can cause bystander damage to epithelial and endothelial cells via proteolytic enzymes, oxidative stress, or histone toxicity, thereby compromising tissue integrity and vascular function. (**B**) **NETs sensing by target cells and amplification of inflammation responses**: Persistent NET components act as DAMPs (Red broken arrows) that are sensed by surrounding immune and non-immune cells. eDNA activates endosomal TLR9 and cytosolic cGAS-STING pathways, leading to IRF3 activation and type I interferon production. NET-associated miRNA engages endosomal TLR7, further promoting interferon signaling. Cit-H3 and HMGB1 can signal through surface TLR2 and TLR4, initiating MyD88-dependent NF-κB activation and robust production of proinflammatory cytokines such as IL-6, IL-1β, and TNF-α. Collectively, these signaling cascades amplify inflammatory immune responses, promote endothelial dysfunction, and sustain neutrophil activation and NET release. Created in Biorender. Njinju Amin Asaba, C. (2026) https://BioRender.com/65mk5g9.

It should be noted that the structural backbone of NET consists primarily of nuclear or mt-DNA, which serves not only as a physical scaffold but also as a potent DAMP. The externalization and persistence of this extracellular DNA (eDNA) in tissues or circulation have been shown to activate innate immune receptors, including endosomal TLR9 and the cytosolic cGAS-STING pathway in multiple cell types, such as macrophages [70], dendritic cells [70], and endothelial cells [71] (see Figure 2). It is well established that the engagement of these pathways can promote the NF-κB and type I interferon signaling, thereby driving sustained cytokine production and amplifying inflammatory responses [71]. In addition to eDNA, other studies have described the presence of NET-associated RNA, including miR-let-7b, a small RNA that complexes with the histone-like alarmin HMGB1 within NETs [72]. This NET-associated miRNA is reported to be expressed at higher levels in LDNs from patients with systemic lupus erythematosus (SLE) and can act as a TLR7 agonist (see Figure 2) to stimulate type I interferon production [72]. Collectively, these findings suggest that NETs function not only as antimicrobial structures but also as immunostimulatory platforms that deliver nucleic acid-based DAMPs to innate immune sensors, thereby perpetuating chronic inflammation and interferon-driven pathology, as seen in some severe viral infections such as SARS-CoV-2 and HBV.

Other NET components, such as citrullinated histone proteins, have been implicated in monocyte activation via the TLR4 pathway. This study demonstrated a synergistic effect between NET DNA and citrullinated histones in enhancing TLR4 recruitment and activation in primary human monocytes [73]. The activation of this pathway initiates downstream signaling events that lead to pronounced production of proinflammatory cytokines such as IL-1β. Moreover, the finding established a direct association between extracellular histones and atherogenesis [73]. Given the increased incidence of atherosclerosis reported in COVID-19 and chronic HBV infections, persistent NET release and histone exposure during chronic viral inflammation may contribute to vascular dysfunction (see Figure 2) and accelerate atherosclerotic disease. In addition to eDNA and citrullinated histones, NETs are also enriched with serine proteases and oxidative enzymes, such as neutrophil elastase (NE), cathepsin-G, proteinase-3, and myeloperoxidase (MPO). While these enzymes play important antimicrobial roles, they can also drive cellular and tissue injury, leading to the release of alarmins [74]. Reports have shown that NE promotes lung tissue injury and facilitates the extracellular release of HMGB1, a key alarmin that acts as a potent ligand for TLRs such as TLR4 [74] (see Figure 2). The engagement of this receptor can trigger inflammatory responses through the NF-κB-dependent signaling pathway.

Taken together, this mechanistic understanding highlights multiple potential therapeutic targets, as modulating these pathways could help mitigate the detrimental effects of dysregulated NET formation. Such interventions may limit excessive innate immune responses, hyperinflammation, tissue injury, and multi-organ dysfunction, as seen in severe cases of SARS-CoV-2 infection and in chronic HBV infection.

## 4. NETs as Drivers of Viral Immunopathology and Post-Virus Syndromes

### 4.1. Immunothrombosis, Endothelial Injury, and Vascular Leakage

**Figure 3:**
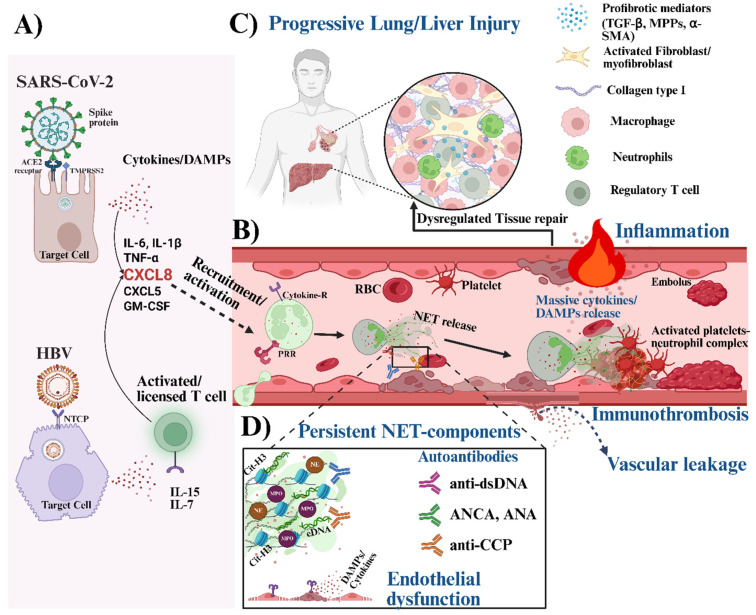
Illustration of NET-induced immunopathology and post-viral sequelae in SARS-CoV-2 and HBV infection. (**A**) **Viral sensing and inflammatory priming:** SARS-CoV-2 and HBV infect target cells through their respective entry receptors (ACE2/TMPRSS2 for SARS-CoV-2 and NTCP for HBV), leading to cellular stress, death, and subsequent release of PAMPs and DAMPs. These signals can mediate the activation of innate and adaptive immune responses and promote the production of proinflammatory cytokines and chemokines, including IL-6, IL-1β, TNF-α, CXCL8, CXCL5, and GM-CSF, as well as T-cell activating/licensing cytokines such as IL-7 and IL-15. This inflammatory milieu promotes the recruitment and activation of neutrophils and other immune cells to the site of infection. (**B**) **Immunothrombosis and vascular injury:** Excessive cytokine and DAMP signaling drives neutrophil activation and extracellular trap release within the vasculature. Persistence of NET components can exert cytotoxic effects on endothelial cells, leading to endothelial activation, apoptosis, and barrier disruption. This endothelial injury promotes plasma leakage into surrounding tissues, triggering platelet recruitment, adhesion, and clot formation. Uncontrolled NET release serves as a scaffold for platelet interaction with neutrophils. This further promotes platelet aggregation, red blood cell trapping, and fibrin deposition, leading to immunothrombosis, microvascular occlusion, endothelial damage, and vascular leakage. These events amplify inflammation and contribute to embolic complications observed in severe viral disease. (**C**) **Hyperinflammation and progressive lung/liver injury:** Excessive tissue damage driven by sustained inflammation and persistent NET components disrupts normal repair mechanisms, promoting maladaptive remodeling in organs such as the lungs (e.g., COVID-19) and liver (e.g., HBV). Neutrophil–macrophage crosstalk promotes fibroblast activation and differentiation into myofibroblasts, leading to the release of profibrotic mediators (e.g., TGF-β, MMPs, α-SMA) and excessive extracellular matrix deposition. These processes culminate in progressive tissue injury and fibrosis, contributing to severe disease phenotypes such as chronic respiratory failure in the lungs or cirrhosis in the liver. (**D**) **NET-induced autoimmune sequelae**: Impaired clearance of NETs results in the persistence of NET-associated molecules (e.g., citrullinated histones, MPO, NE, eDNA) that act as DAMPs and autoantigens. These components induce endothelial dysfunction and promote the generation of autoantibodies, including anti-dsDNA, ANCA, ANA, and anti-CCP. Collectively, persistent NETosis links acute viral inflammation to long-term autoinflammatory and autoimmune sequelae, contributing to post-viral syndromes and chronic organ dysfunction. Created in Biorender. Njinju Amin Asaba, C. (2026) https://BioRender.com/es4whyb.

Studies have shown that certain viral infections can drive the dysregulation of neutrophil functions, leading to an excessive release and persistence of NET components such as citrullinated histones or proteolytic enzymes (cathepsin-G, NE, and proteinase-3) that can exert cytotoxic effects on endothelial cells, leading to endothelial activation, apoptosis, and barrier disruption [17,75]. This resulting endothelial injury can compromise the vascular integrity and promote plasma leakage into surrounding tissues [75] (see Figure 3). Collectively, these processes can exacerbate local inflammation and tissue hypoxia, fueling a self-amplifying cycle of vascular damage and immunothrombosis [75]. Notably, in severe COVID-19, such dysregulated NET formation has been associated with endothelial dysfunction, sepsis-like pathology, and autoimmune vasculitis [75,76,77].

Severe SARS-CoV-2 infection is characterized by a complex interplay between platelets and neutrophils that amplifies thrombo-inflammatory responses in the pulmonary vasculature [76,78,79] (see Figure 3). It should be noted that activated platelets could directly interact with neutrophils through P-selectin and PSGL-1 ligand engagement. This interaction can enhance NET release and further platelet activation [79]. In connection with this, clinical evidence indicates that the severity of COVID-19 correlates with elevated platelet-neutrophil aggregate counts, which contribute to microvascular thrombosis and endothelial injury in lung tissue [80,81]. This observation is further supported by elevated levels of soluble markers of thrombosis, such as plasma D-dimer and von Willebrand factor (VWF) antigen, in COVID-19 patients compared with healthy individuals [81,82]. In addition, microvascular thrombi containing neutrophils that actively release NETs and are mixed with platelets have been reported in lung biopsies from deceased COVID-19 patients [83,84]. This highlights the central role of dysregulated NETosis in COVID-19-associated immunothrombosis and its contribution to respiratory failure and mortality.

In the context of chronic HBV infection, severe complications such as portal vein thrombosis (PVT) have been reported, particularly among patients with advanced liver cirrhosis [85,86]. Although the precise mechanisms remain to be fully elucidated, it could be hypothesized that the elevated neutrophil levels observed in patients with severe HBV [25] may promote platelet activation and interaction, thereby favoring endothelial injury and fibrin deposition within the portal venous system. Moreover, studies have shown that PVT patients often present with elevated plasma levels of NET components, such as cell-free DNA and histone-DNA complexes, which are potential risk factors for overall disease outcome [87]. Therefore, a better understanding of the molecular interplay between activated neutrophil-platelet interactions could provide novel insights into the pathogenesis of HBV or SARS-CoV-2 associated thrombosis and identify potential therapeutic strategies targeting NET-mediated immunothrombosis.

### 4.2. Neutrophils in Hyperinflammation and Progressive Lung/Liver Injury

Severe viral infections often trigger a strong inflammatory response that begins at the level of infected epithelial or endothelial cells. During entry or replication, viral structural components could serve as PAMPs or as triggers for DAMP release from dying cells [88]. These signals could be detected by a variety of surface, endosomal, or cytosolic sensors expressed by infected or uninfected cells to trigger the induction of distinct inflammatory response pathways, including the RIG-I [88], MDA5 [88], AP-1 [89], NF-κB, or inflammasome [88,90]. In severe SARS-CoV-2 infection, these pathways are highly activated, leading to excessive production of proinflammatory mediators such as IL-6, IL-1β, TNF-α, CXCL8, CXCL10, CXCL1, CXCL5, and GM-CSF [15,37,89]. The uncontrolled amplification of these pathways often culminates in the so-called cytokine storm, which not only reflects a systemic immune dysregulation but also promotes extensive leukocyte recruitment to infected tissues [89]. Among the recruited cells, neutrophils are markedly elevated, particularly in the lungs, as seen in severe COVID-19 patients [15,91]. These cells accumulate in large numbers in response to excessive chemoattractants (e.g., CXCL8, CXCL5, CXCL1) released by infected epithelial cells, activated macrophages, and damaged endothelial cells [92,93].

The high neutrophil influx brings them into frequent contact with viral particles or DAMPs released from damaged cells, thereby increasing their likelihood of excessive NET formation [94,95] (see Figure 3). It has been reported that impaired clearance mechanisms, due to reduced DNase activity, an overwhelming NET burden, or macrophage exhaustion, allow NET components to persist within tissues [24,27]. The accumulation of uncleared NETs serves as a potent source of DAMPs that can engage PRRs such as TLR9, TLR7, TLR2, and TLR4 on macrophages and other immune cells, as well as on stromal cells [70,72,73]. This amplifies local inflammation through excessive release of proinflammatory cytokines and perpetuates tissue injury, often requiring the immune system to activate robust repair mechanisms. Even though this pro-resolving phase is essential after viral clearance, it is often incomplete or dysregulated. When cells responsible for clearing debris, extinguishing inflammation, and restoring tissue homeostasis function inefficiently, low-grade inflammation can persist and drive ongoing hepatocellular injury, ultimately promoting maladaptive wound-healing responses and, in some individuals, progressive fibrosis. Macrophages are key regulators of this transition, largely through the release of anti-inflammatory cytokines, growth factors, or pro-resolving mediators that facilitate tissue repair and restore homeostasis [96]. However, studies have shown that even after viral clearance, lung tissue analyses of severe COVID-19 patients revealed the presence of several NET components and an altered macrophage phenotype, with increased foam cell formation and expression of profibrotic markers [97,98]. This therefore suggests that persistent inflammatory cues driven by the sustained presence of NET components, along with defective efferocytosis or maladaptive metabolic reprogramming of macrophages, could initiate an aberrant repair response that promotes fibroblast activation and excessive extracellular matrix deposition. Ultimately, this dysfunctional macrophage state may contribute to persistent inflammation and maladaptive tissue repair, potentially promoting fibrotic remodeling and longer-term respiratory impairment in some individuals after severe COVID-19 [99].

The destructive effects of specific NET components, including their contribution to tissue fibrosis, are increasingly recognized. One such component is the nucleus-resident proinflammatory factor high-mobility group box protein 1 (HMGB1). Although HMGB1 is known for its protective roles during viral infections, growing evidence suggests that it may also participate in SARS-CoV-2 induced lung fibrosis [100]. In addition to activating inflammatory signaling through the TLR2/4 or RAGE receptor axis, HMGB1 has been implicated in driving fibrotic responses in certain pathological settings [101]. While the precise mechanisms linking HMGB1 to fibrosis in COVID-19 remain unclear, in vitro studies demonstrate that HMGB1 can stimulate human lung fibroblasts to produce profibrotic mediators such as TGF-β1 and type I collagen via NF-κB activation [101]. Moreover, patients with severe COVID-19 display elevated levels of immature low-density neutrophils that release abundant NETs enriched in HMGB1-DNA complexes [102]. These complexes may potentiate fibroblast activation, sustain NF-κB-driven cytokine production, and create a microenvironment that favors extracellular matrix deposition and tissue remodeling, thereby linking excessive NET formation to the progression of lung fibrosis or respiratory failure in severe SARS-CoV-2 infection (see Figure 3). In addition, studies have also described the involvement of other key NET components, such as MPO and histone proteins, in promoting fibroblast activation and differentiation into myofibroblasts, a cell type marked by the expression of profibrotic factors such as α-SMA, collagen I, and other ECM proteins [103].

Although the onset of lung tissue fibrosis is multifactorial, accumulating reports point to a central role for neutrophil–macrophage crosstalk in orchestrating this pathological transition. As patients progress from early hyperinflammation to the formation of fibrotic lesions, the overwhelming neutrophil–macrophage dysregulated responses become prominent features. It has been suggested that SARS-CoV-2-induced acute lung injury (ALI) can accelerate the onset of pulmonary fibrosis, ultimately predisposing individuals to respiratory failure and increased mortality [104]. Mechanistically, studies in fibrotic lung disease, including COVID-19-associated fibrosis, have demonstrated that neutrophils frequently colocalize with SPP1^+^ profibrotic macrophages, forming a spatially organized niche that reinforces fibrogenic signaling [105]. Within this niche, neutrophils secrete matrix metalloproteinases (MMPs), particularly MMP8 and MMP9, which facilitate extracellular matrix remodeling and proteolytically activate latent TGF-β1 within the ECM [105]. The resulting increase in active TGF-β1 enhances fibroblast migration and drives their differentiation into profibrotic myofibroblasts, ultimately perpetuating a self-sustaining cycle of inflammation and fibrosis (see Figure 3). With a growing body of evidence demonstrating elevated SPP1^+^ macrophage signatures in patients with severe COVID-19 [106,107], it is tempting to speculate that enhanced neutrophil–macrophage interactions may create a fibrosis-permissive microenvironment that accelerates the transition from acute lung injury to chronic fibrotic remodeling. Similarly, clinical data from hepatitis B virus-related acute-on-chronic liver failure (ACLF) patients, especially those with decompensated liver cirrhosis, show elevated neutrophil numbers with aberrant functions [25]. Neutrophils from these patients produced excessive amounts of NETs with decreased phagocytic capacity. There was also a positive correlation between increased absolute neutrophil counts and the risk of death in patients with cirrhosis. In addition, transcriptomic analysis revealed significant expression of genes associated with neutrophil degranulation, trap formation, and extracellular matrix remodeling (notably MMP8/9) among these ACLF patients [25]. Although the authors did not associate this study with macrophages, other studies have confirmed the presence of similar M2-like macrophages during acute/chronic HBV infection that localize within fibrotic regions and correlate with liver disease severity/failure [108], mirroring closely what has been reported in severe COVID-19 [109,110]. These findings suggest that neutrophil–macrophage crosstalk may represent a pathogenic axis that promotes the transition from acute inflammation to maladaptive fibrotic remodeling across distinct organs such as the lungs and liver. Altogether, this interaction could serve as a molecular switch that reshapes cellular phenotypes and acts as a potent amplifier of fibroblast activation and extracellular matrix deposition, thereby accelerating the progression toward irreversible tissue fibrosis and organ dysfunction. Therefore, it would be imperative to elucidate the specific neutrophil–macrophage interactions and mediators that drive this profibrotic process, as these may open new avenues for therapeutic targets that halt or reverse fibrosis across multiple inflammatory viral diseases.

### 4.3. Autoinflammatory and Autoimmune Sequelae

Severe viral infections are often accompanied by a cytokine storm, which is a pathological state driven by the excessive release of proinflammatory cytokines as the virus overwhelms the host immune system [15,89,111]. This phenomenon became particularly evident during the COVID-19 pandemic, where severe SARS-CoV-2 infection frequently induced hyperinflammatory states that contributed to acute respiratory distress syndrome (ARDS), multi-organ dysfunction (MOD), or long-term immune dysregulation. Central to this effect is the innate immune system, whose rapid, non-specific responses can be pathologically amplified during severe viral infections [92,112]. Moreover, numerous clinical reports highlight the prominent role of neutrophils and macrophages in driving the excessive release of proinflammatory cytokines such as IL-6, IL-1β, and TNF-α during severe SARS-CoV-2 infection [113]. This resulting inflammatory milieu not only causes acute tissue damage but also sets the stage for the onset of autoinflammatory and autoimmune sequelae, including persistent systemic inflammation, autoreactive immune responses [18,59], and post-viral syndromes observed in conditions such as multisystem inflammatory syndrome, or what is now widely described as long COVID [18].

Autoinflammatory diseases are often distinguished by transient pathological responses of the innate immune system, in contrast to the often-permanent autoimmune diseases driven by an autoreactive adaptive immune system. In severe COVID-19, the overwhelming activation of innate immune cells can trigger a hyperinflammatory cascade that mimics autoinflammatory pathology [106]. When sustained, this dysregulated inflammatory state may drive the production and systemic release of various cell- or tissue-associated molecules that the immune system recognizes as foreign, thereby providing immunogenic signals that break immune tolerance and promote the emergence of autoreactive lymphocytes [18]. Consequently, severe SARS-CoV-2 infection can give rise to a spectrum of downstream immune dysregulation, ranging from acute autoinflammatory-like episodes to long-term autoimmune sequelae [18,59,102]. As described earlier, severe COVID-19 patients often present with dysregulated neutrophil functions, including increased NET formation. The spontaneous release and persistence of NET components can deliver a variety of self-modified molecules that are perceived as foreign by the immune system (see Figure 3). Perhaps, with existing evidence showing that many of the molecules externalized through NET formation, such as MPO, eDNA, cathelicidin, and citrullinated histones, can be recognized as autoantigens [102]. It is plausible to suggest that aberrant NET formation during SARS-CoV-2 infection may play an important role in initiating autoimmune responses in susceptible individuals.

Autoimmune sequelae have been clinically documented following viral infections, with COVID-19 providing one of the most comprehensive examples [3,18,59]. Post-acute COVID-19 or long COVID patients have demonstrated an increased incidence of autoimmune diseases, including systemic lupus erythematosus (SLE) [114], rheumatoid arthritis (RA) [115], multiple Sclerosis (MS) [116], autoimmune thyroiditis [117], type 1 diabetes [118], ANCA (anti-neutrophil cytoplasmic antibodies)-associated vasculitis (AAV) [18], and polymyositis [119]. Many of these conditions have been linked to neutrophil hyperactivation, excessive NET release, and heightened inflammatory signatures [18]. Given the distinct pathogenic mechanisms across these disorders, it is plausible that their manifestations in long COVID patients could depend on individual susceptibility and the degree of persistent exposure to different NET components. The development of SLE-like or RA-like phenotypes after COVID-19 further supports the hypothesis that viral infections can act as environmental triggers of systemic autoimmunity.

Consistent with this, several reports have shown that poor prognostic outcomes in COVID-19 are often associated with increasing neutrophil numbers, exhibiting a characteristic low-density phenotype. These cells synthesize more proinflammatory cytokines and have an enhanced capacity to form NETs, while maintaining increased cellular viability. It has been demonstrated that these NETs generated by LDNs contain various molecules, such as citrullinated proteins, cathelicidin-DNA complexes, and oxidized nucleic acids, which can function as autoantigens [13,23,120]. When these NET-derived molecules persist in the bloodstream, they can trigger the production of antibodies against them (see Figure 3). This process is linked to higher serum levels of anticyclic citrullinated peptide [120] or anti-dsDNA antibodies [120], thereby supporting a direct contribution of NET formation to the pathogenesis of RA or SLE, respectively. Moreover, clinical data have revealed elevated circulating antibodies against NET-associated molecules (including ANAs, anti-dsDNA, ANCA, antiphospholipid antibodies, and anti-CCP) in patients with COVID-19 [114,115,117]. These observations make it increasingly evident that SARS-CoV-2 infection can potentiate systemic autoimmunity in susceptible individuals. Collectively, these findings support a hypothesis that excessive or dysregulated NET formation not only drives COVID-19 severity but also serves as a mechanistic link between severe viral infection and the initiation or exacerbation of autoimmune diseases such as AAV, myositis, RA, and SLE.

The prevalence of symptoms resembling RA and SLE has been increasingly reported in individuals with long COVID [120]. In fact, evidence indicates that interferon (IFN)-driven pathways may contribute to the immunopathogenesis of long COVID, paralleling key features of SLE [121]. Recent data demonstrate coordinated elevation of multiple IFN subtypes, including IFN-α2, IFN-β, IFN-γ, and type III IFNs, in patients with long COVID, with biological IFN activity strongly correlating with serum anti-dsDNA levels [121]. Given that anti-dsDNA antibodies typically arise following sustained exposure to extracellular chromatin, these findings suggest that persistent eDNA potentially derived from excessive NET formation may act as a continuous endogenous trigger of IFN signaling. Mechanistically, NET-derived eDNA can be sensed by endosomal DNA receptors such as TLR9, promoting robust type I IFN production [70]. Concurrently, prolonged exposure to eDNA may drive the generation of anti-dsDNA antibodies, leading to immune complex formation that further amplifies IFN signaling. SARS-CoV-2 infection, which induces strong interferon responses [122,123], may therefore create a permissive environment in which excessive NET formation and impaired clearance sustain an eDNA-IFN amplification loop. In genetically predisposed individuals, persistent activation of this interferon axis may lower the threshold for immune tolerance breakdown and promote lupus-like symptoms as seen in long COVID. Furthermore, the excessive release of activated PAD4 during NET formation can drive extensive citrullination and destabilization of self-proteins, altering their structure in ways that render them immunologically foreign, thereby promoting their recognition as autoantigens and triggering autoimmune responses [124]. This mechanism is particularly relevant in RA, where citrullinated proteins constitute major autoantigens targeted by anti-citrullinated protein antibodies (ACPAs). Viral infections such as SARS-CoV-2 may therefore contribute to RA pathogenesis by enhancing NET-driven citrullination and expanding the pool of neoantigens available for immune recognition. Collectively, these processes provide a plausible mechanistic framework linking virus-induced NET dysregulation to the autoimmune features increasingly observed following SARS-CoV-2 infection, including long COVID [18].

Similarly, in other viral infections, such as HBV, the presence of autoantibodies and a range of autoimmune manifestations has been documented [26,125,126]. However, key insights into the immunopathological mechanisms driving HBV-associated autoimmunity remain considerably less well defined. In particular, whether HBV triggers neutrophil activation and NET-driven autoantigen exposure to the same extent as SARS-CoV-2 is still unclear. Additionally, the contribution of NETs to HBV-related autoimmune sequelae remains unclear.

Therefore, it would be valuable to further investigate how prolonged neutrophil dysfunction, specifically hyperactivation of LDNs and excessive NET formation, may serve as a pathomechanistic bridge between viral infections and the development of autoimmune diseases such as SLE and RA. While this connection is increasingly well-supported in COVID-19, similar questions remain largely unexplored in the context of HBV, where reports of vasculitis, RA, and other autoimmune features suggest that NET-mediated pathways may also contribute to disease pathogenesis [127,128]. Since NETs contain various molecules that can function as autoantigens, a deeper understanding of the pathways leading to NET formation, citrullination, interferon amplification and the subsequent production of autoantibodies in both SARS-CoV-2 and HBV infections could be essential for developing targeted therapeutic strategies.

## 5. Therapeutic Strategies Targeting NET Dysregulation in Viral Infections

### 5.1. Targeting NET Formation

The recognition that dysregulated NET formation plays a significant role in the immunopathology of viral infections has prompted several initiatives to develop innovative therapeutics. However, the dual function of NETs as antimicrobial agents may contribute to tissue injury, presenting a considerable challenge for their modulation. Therapeutic interventions must therefore carefully balance the minimization of harmful inflammation associated with NET formation with the preservation of host immune function. Several studies have identified some molecular targets as potential therapeutic options, with each of the targets characterized by their distinct mechanisms and clinical outcomes, as detailed below:

#### 5.1.1. Peptidylarginine Deiminase 4 (PAD4)

**Figure 4:**
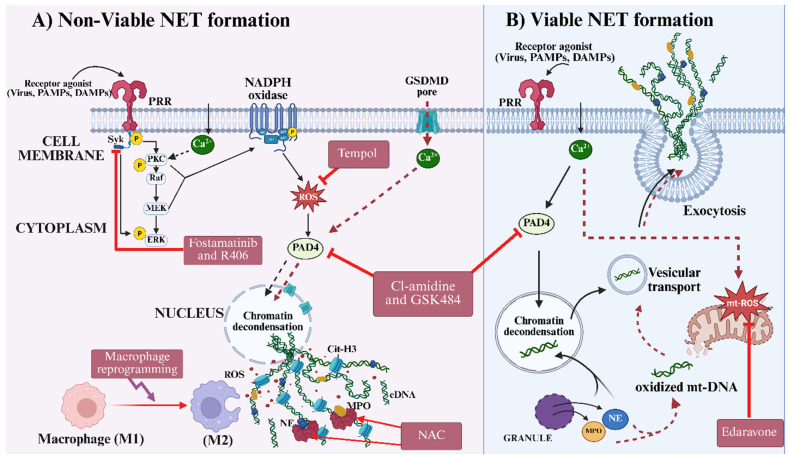
Therapeutic approaches to target the different mechanisms of non-viable (**A**) and viable NET formation (**B**). NETs can be modulated by targeting their formation, for example, by inhibiting Peptidylarginine deiminase 4 (PAD4) with therapeutics such as CL-amidine and CSK484 and also by using reactive oxygen species (ROS) scavengers such as NAC, which can inhibit myeloperoxidase (MPO) and neutrophil elastase (NE); tempol, which reduces the oxidative burst required for NET formation; and edaravone, which suppresses mitochondrial ROS generation, all leading to the inhibition of NET formation. Furthermore, therapeutics targeting Spleen tyrosine kinase (SYK) and mitogen-activated protein kinase (MAPK) inhibitors, such as Fostamatinib and R406, also inhibit the initiation of NETs. Other therapeutic approaches focus on enhancing NET clearance, such as the use of annexin A1, glucocorticoids, and specialized pro-resolving lipid mediators, including resolvins and protectins, which promote M2 macrophage polarization, enhance efferocytic capacity, and thus clear NETs. Created in Biorender. Ayuk, S. (2026) https://BioRender.com/7qbyt27.

PAD4 is a calcium-dependent enzyme expressed in the cytoplasm of most cells and is essential for NET formation. It catalyzes the citrullination of histones, such as H3 and H4, thereby facilitating chromatin decondensation, a necessary step for NET release [129,130,131]. Therefore, inhibiting PAD4 offers a potential therapeutic option for preventing NET formation and protecting against organ injury. Recent studies have shown that a deficiency in PAD4 alleviates lung injury and airway inflammation in models of chronic obstructive pulmonary disease [132,133]. Moreover, certain pharmacological compounds, such as Cl-amidine and GSK484, which function as PAD4 inhibitors (see Figure 4), have shown protective effects in animal models of sepsis, autoimmune disorders, and cancer by suppressing NET formation through the blockade of histone citrullination [134,135]. Furthermore, it was also observed that by preventing PAD4-driven fibroblast activation and endothelial damage, PAD4 inhibitors could suppress pathological fibrosis and limit metastatic progression in cancer [136,137]. Therefore, PAD4 inhibition offers significant therapeutic potential by preventing histone citrullination, thereby diminishing NET-mediated vascular damage and thrombosis.

Although PAD4 inhibition provides a potential therapeutic option, a significant drawback might be a compromise in NETs’ antimicrobial defense mechanisms. NETs are crucial components of the innate immune system and are important for capturing and neutralizing bacteria, fungi, and viruses [138,139,140]. Inhibition of PAD4 and, consequently, NET formation may increase susceptibility to infections, particularly sepsis or pneumonia [141,142]. Thus, while PAD4 inhibition offers an effective means of controlling NET-induced inflammation and tissue damage, its application, however, requires an optimal balance between immune protection and disease susceptibility.

#### 5.1.2. Reactive Oxygen Species (ROS) Scavengers

ROS generated by NADPH oxidase (NOX) (NOX-dependent pathway) or mitochondrial (NOX-independent pathway) function as a crucial signaling molecule for the induction of neutrophil extracellular traps (NETs) [143,144]. By targeting either NADPH oxidase or mitochondria-derived ROS, certain scavengers, such as N-acetylcysteine (NAC), tempol (4-hydroxy-2,2,6,6-tetramethylpiperidine-N-oxyl), and edaravone (3-methyl-1-phenyl-2-pyrazolin-5-one), have been shown to inhibit NET formation, histone citrullination, and neutrophil degranulation.

N-acetylcysteine (NAC) was first approved in 1963 by the U.S. Food and Drug Administration for the treatment of respiratory diseases [145]. Being a cysteine precursor, NAC has been reported to reduce NET formation by replenishing glutathione (GSH), the principal cellular antioxidant [146,147]. In addition, NAC scavenges hydrogen peroxide and other peroxides generated during the NADPH oxidase-dependent oxidative burst, thereby inhibiting the activation of myeloperoxidase (MPO) and neutrophil elastase (NE) (see Figure 4), two enzymes critical for chromatin decondensation and NET release [147,148]. Through these mechanisms, NAC not only suppresses NET formation but also reduces circulating NET components and inflammatory cytokines in autoimmune diseases, such as lupus and rheumatoid arthritis [149].

Tempol (4-hydroxy-2,2,6,6-tetramethylpiperidine-N-oxyl) is a membrane-permeable piperidine nitroxide with a low molecular weight and superoxide dismutase (SOD) mimetic [150]. It has been reported to inhibit NET formation by catalytically converting superoxide anions (O_2_•^−^) into less reactive species, thereby attenuating the oxidative burst required for NET formation [151,152,153]. By reducing intracellular superoxide and peroxynitrite levels, the oxidative activation of MPO and PAD4, two downstream mediators of histone modification and chromatin expansion, is suppressed. This antioxidant effect thus stabilizes neutrophil membranes and limits NET formation, thereby regulating inflammation and tissue damage. In animal models, tempol has been shown to effectively reduce vascular NET accumulation, thrombosis, and endothelial dysfunction, highlighting its therapeutic potential in an oxidative stress-driven vascular and autoimmune inflammation [150,151,154].

Edaravone (3-methyl-1-phenyl-2-pyrazolin-5-one) is a potent free radical scavenger that has been used to treat acute brain infarction [155]. It prevents NET formation by neutralizing hydroxyl radicals and suppressing mitochondrial ROS generation [156] (see Figure 4). Also, it protects mitochondrial integrity and prevents the oxidative activation of histone-modifying enzymes, such as PAD4, thereby blocking chromatin decondensation [157]. This mechanism points to the therapeutic benefits of Edaravone in reducing NET-driven vascular and neuronal damage in stroke and ischemia–reperfusion injury models [157,158].

ROS scavengers, therefore, offer anti-inflammatory and tissue-protective effects similar to those of PAD4 by restoring redox balance. However, since ROS production is central to neutrophil antimicrobial activity, scavenging its products might present a risk of impairing innate immune defense mechanisms, as observed in some studies [159,160]. This is because excessive ROS scavenging can impair the oxidative burst required to kill pathogens, thereby predisposing the host to infections by bacteria, fungi, and viruses. As with PAD4 inhibition, ROS scavengers require strategic therapeutic application to dampen the pathological effects of NETosis without entirely abolishing the host’s antimicrobial capacity.

#### 5.1.3. Spleen Tyrosine Kinase (SYK) and Mitogen-Activated Protein Kinase (MAPK) Inhibitors

The SYK and MAPK pathways are integral in transmitting essential signals from pattern recognition receptors and cytokine receptors, thereby initiating NET formation [54,161,162]. SYK functions downstream of various receptors, including integrins, Fc receptors, TLRs, and C-type lectins, and it phosphorylates downstream effectors that lead to NADPH oxidase activation and cytoskeletal rearrangement [161]. Similarly, the MAPK signaling pathway, encompassing ERK1/2, JNK, and p38 kinases, regulates inflammatory gene expression and post-translational modifications crucial for NET formation [163]. Inhibition of these kinases has shown potential to prevent the pathological effects of NET formation. For example, SYK inhibitors, such as fostamatinib and R406, were reported to block NET induction effectively [164] (see Figure 4). Likewise, p38 MAPK inhibitors diminish NET formation in response to inflammatory cytokines and viral infections [165,166]. The advantage of these kinase inhibitors is their ability to modulate cellular activation states that are vital for NET formation without excessive inflammation and to preserve innate antimicrobial functions. For instance, in viral infections characterized by hyperinflammation and NET-mediated complications, SYK and MAPK inhibitors can offer targeted immunomodulation [167]. However, SYK and MAPK signaling are also involved in adaptive immune responses, including B cell and T cell activation, vital for long-term viral control and immunological memory [168,169,170]. Thus, prolonged inhibition of SYK and MAPK might compromise pathogen clearance and antibody production. Therefore, it is important to consider the dosage and duration of SYK and MAPK inhibition to avoid impairing pathogen clearance and adaptive immune responses.

### 5.2. Enhancing NET Clearance

Enhancing NET clearance constitutes a complementary therapeutic strategy to inhibiting their formation. This approach acknowledges that while NETs may initially confer benefits, their persistence in tissues or circulation can cause tissue injury. The degradation and removal of NET components are critical homeostatic processes; when impaired, these processes lead to the accumulation of prothrombotic and proinflammatory chromatin complexes in the bloodstream. To effectively clear NET products, two primary approaches have been identified: enzymatic degradation of the DNA scaffold using exogenous nucleases and the restoration of macrophage-mediated phagocytic clearance (efferocytosis).

#### 5.2.1. Deoxyribonuclease I (DNase I) Therapy

DNase I is an endonuclease that catalyzes the hydrolysis of extracellular DNA, thereby degrading the structural backbone of NETs and releasing trapped proteins [171]. Studies have shown that in pathological conditions such as lupus, sepsis, and acute lung injury, impaired DNase activity results in NET accumulation, endothelial injury, and autoantigen exposure [172,173,174]. Recombinant human DNase I (dornase alfa), currently used in cystic fibrosis treatment, has demonstrated efficacy in degrading NETs, reducing mucus viscosity, and improving lung function [175,176]. Furthermore, preclinical studies have shown the benefits of DNase I treatment in models of viral pneumonia. In COVID-19 patients, DNase treatment ameliorated microvascular thrombosis and respiratory distress syndrome [177,178]. However, DNase I therapy presents significant practical and biological limitations. The enzyme exhibits suboptimal stability in certain clinical applications [179]. Consequently, achieving adequate tissue penetration, particularly in consolidated lung parenchyma or thrombosed vasculature, poses pharmacokinetic challenges. Furthermore, DNA degradation releases nucleosomes and smaller chromatin fragments that may possess inflammatory and thrombogenic properties [180], potentially triggering a proinflammatory state.

#### 5.2.2. Macrophage Reprogramming

Beyond enzymatic degradation, macrophage reprogramming significantly enhances NET clearance via efferocytosis [181]. Macrophages identify NETs via various receptors that bind to histones, DNA, and granular proteins, and subsequently internalize and degrade these structures within phagolysosomes. This process is predominantly anti-inflammatory, as macrophages engaged in efferocytosis typically assume an M2-like phenotype (see Figure 4), characterized by increased secretion of IL-10, TGF-β, and other immunoregulatory mediators [182]. However, in severe viral infections, macrophages’ clearance capacity is often impaired because proinflammatory cytokines can polarize macrophages towards the M1 phenotype, thereby limiting their clearance capacity [183,184]. Additionally, viruses may directly infect and impair macrophage function, thereby inhibiting NET clearance [185]. Therapeutic strategies involving the administration of agents that promote M2 polarization or enhance efferocytic capacity, such as annexin A1, glucocorticoids, or specialized pro-resolving lipid mediators, including resolvins and protectins, are an effective approach towards NET clearance [186]. These molecules upregulate phagocytic receptors, enhance phagolysosomal processing, and suppress inflammatory signaling in macrophages. Thus, the combination of DNase I therapy with macrophage reprogramming represents a potential synergistic approach that enzymatically dissolves NET structures, re-establishes innate immune balance, and prevents tissue damage.

### 5.3. Risk-Benefit Assessment and Timing in NET-Targeted Therapies

As mentioned earlier, therapeutic modulation of NETs presents a fundamental clinical challenge: NETs provide essential early antimicrobial defense but can also contribute to tissue injury, thrombosis, and organ dysfunction when persistent. Therefore, the safety and efficacy of NET-targeted interventions should critically depend on disease stage, therapy duration, and the consequences of NET inhibition. For example, PAD4 [136,137] and ROS scavengers [145,151] suppress NET-driven inflammation and tissue damage, but can also simultaneously compromise the antimicrobial function of NETs in the long run. Thus, their inhibition should therefore be restricted to brief therapeutic windows during the hyperinflammatory phase. Regarding SYK and MAPK, which are vital for B-cell and T-cell activation [168,169,170] in controlling viral infection and long-term immunity, their inhibition should also be limited to brief periods of acute hyperinflammation. In addition, their use should be guided by biomarkers of NET formation (such as citrullinated histone H3 or MPO–DNA complexes) to delineate their initiation and discontinuation. In DNase I-based therapeutic strategies, degradation products can activate Toll-like receptors, thereby perpetuating inflammatory signaling [187]. Therefore, DNase therapy may be most effective when combined with other therapeutic strategies, such as macrophage reprogramming, to prevent the accumulation of inflammatory degradation products. Collectively, these considerations underscore the need for a precision-based approach in NET-targeted therapies that balances antimicrobial defense with the prevention of NET-mediated tissue injury.

### 5.4. Lessons from COVID-19 and Hepatitis B Virus Clinical Trials

The COVID-19 pandemic has yielded significant insights into the potential of NET clearance as a therapeutic target. Patients with severe SARS-CoV-2 infection presented excessive NET accumulation in the lungs and vasculature, contributing to thrombosis, acute respiratory distress syndrome (ARDS), and multiorgan failure [188,189,190]. Moreover, an increase in NET biomarkers, such as citrullinated histone H3, cell-free DNA, and MPO-DNA complexes, has been reported in the serum of patients with COVID-19 [94]. In addition, COVID-19 patients with severe disease outcomes exhibited reduced DNase activity and impaired macrophage-mediated NET degradation, correlating with hypercoagulability and cytokine storm [59,191,192]. Clinical trials utilizing inhaled dornase alfa demonstrated reductions in sputum NET content, improved oxygenation, and decreased inflammatory markers in hospitalized patients [193,194,195]. Preliminary results from ongoing clinical trial studies have further suggested that DNase I treatment may shorten hospital stay and reduce mechanical ventilation needs when combined with standard corticosteroid therapy (NCT04359654) [193]. Thus, in the context of COVID-19, studies on therapeutic strategies that combine DNase I administration with macrophage reprogramming agents (such as IL-6 blockers or resolvins) may enhance NET clearance while maintaining immune response.

HBV infection also offers unique therapeutic approaches to modulate the interplay among NET formation, antiviral immunity, and tissue injury. It has been shown that although NETs can entrap viral particles and limit viral dissemination, excessive NETs in chronic HBV infection [7] can, however, contribute to hepatic inflammation, fibrosis, and hepatocyte injury [7,196,197]. As with COVID-19, patients with active HBV infection have elevated levels of NET-associated biomarkers, such as cell-free DNA and myeloperoxidase-DNA (MPO-DNA) complexes, in their peripheral blood samples [26]. Surprisingly, there are currently no clinical trials specifically targeting NET formation in HBV infection. However, other clinical trial results have shown that Entecavir and Tenofovir disoproxil fumarate are currently recommended for the treatment of chronic hepatitis B due to their inhibition of viral replication [198,199]. Therefore, novel therapeutic strategies that incorporate NET clearance, as mentioned above, in combination with Entecavir and Tenofovir could reduce both viral replication and immune-mediated hepatocellular injury, offering a more comprehensive approach to managing chronic HBV.

## 6. Conclusions and Future Perspectives

Neutrophil extracellular traps (NETs) have emerged as central players at the intersection of innate immunity, hyperinflammation, and tissue injury or multi-organ dysfunction during viral infections. In recent years, our understanding of neutrophil immunobiology has expanded far beyond their classical roles in phagocytosis and degranulation, recognizing NET formation as a highly dynamic and tightly regulated antimicrobial mechanism. As discussed throughout this review, NET formation is a double-edged sword, contributing to both antiviral immunity and immunopathology, including hyperinflammation, immunothrombosis, fibrosis, and autoimmune sequelae. Severe or chronic viral infections such as SARS-CoV-2 and HBV exemplify how this delicate balance can be disrupted, resulting in both acute and long-term disease or pathological consequences.

A critical appreciation of the current evidence is that viruses do not merely coincide with NET release and subsequent NETosis as passive triggers; instead, they actively modulate neutrophil signaling and the inflammatory microenvironment to promote, skew, or restrain NET generation, by viable NET release and/or non-viable (lytic) NETosis through virus-specific mechanisms. Different viral components can directly engage neutrophil pattern-recognition receptors, such as TLRs, CTLRs, or Fc receptors, to activate signaling pathways involving SYK, PKC, MAPKs, NF-κB, and ROS generation, culminating in NET release. At the same time, certain viruses have evolved different strategies to evade or exploit NETs, either by suppressing NET formation, degrading NET components, or hijacking NET-associated molecules to enhance viral attachment and infectivity. Therefore, this evolutionary arms race underscores the importance of NETs in antiviral defense while also highlighting how their dysregulation and persistence can promote viral persistence and tissue or organ injury.

Another major aspect highlighted by studies described in this review is the effect of impaired NET clearance in sustaining inflammation and driving chronic pathology. The removal of NETs through DNase-mediated degradation and effective macrophage efferocytosis is crucial for the resolution of inflammation and restoration of tissue homeostasis. Failure in these processes leads to the accumulation and persistence of NET-derived DAMPs, including eDNA, citrullinated histones, proteases, and nucleus-derived alarmins such as HMGB1 [17,72]. Uncleared or persistent NET components activate innate immune receptors, such as TLRs and the cGAS-STING axis, thereby sustaining the NF-κB and type I interferon signaling pathways. This self-amplifying inflammatory cycle provides a mechanistic framework for understanding how acute viral infections can transition into chronic inflammatory states, tissue fibrosis, and post-viral syndromes.

Notably, emerging evidence indicates that neutrophil heterogeneity is a critical determinant of these pathological outcomes. With severe viral infections marked by an increased expansion of immature or LDNs subsets and exaggerated NET formation [15,63,87], it is not unexpected that these aberrant neutrophil populations play a central role in driving persistent inflammation and immune dysregulation. Whether these immature neutrophil populations arise from emergency hematopoiesis triggered by overwhelming viral infection or from altered maturation cues in the bone marrow remains an active area of investigation. What is evident is that this population of neutrophils exhibits prolonged survival and resistance to clearance, enabling them to persist as drivers of inflammation and sources of autoantigens [24,60,61,128], thereby linking innate immune dysregulation to adaptive immune activation and loss of tolerance. Supporting this concept, studies in models of alcohol-induced hepatitis have revealed an accumulation of clearance-resistant, defective low-density neutrophils that exacerbate liver injury and markedly increase susceptibility to sepsis, highlighting a conserved pathogenic role for these dysfunctional neutrophil subsets across inflammatory molecular contexts [24,60]. Collectively, these findings point to a unifying framework that may explain the increasing reports of autoimmune and autoinflammatory diseases following severe viral infections, including long COVID and autoimmune hepatitis. Therefore, understanding the molecular signals that govern the expansion and activation of these neutrophil subsets is crucial, as it may reveal therapeutic targets to mitigate hyperinflammation without compromising antiviral defense.

From a translational perspective, targeting NET formation or NET components may be both attractive and challenging therapeutic strategies. Excessive NETs contribute to tissue damage and exacerbate disease pathology, making them a clear therapeutic target. At the same time, NETs play a crucial role in host defense against pathogens, so broad inhibition risks compromising antimicrobial immunity. Although the different strategies employed to limit NET formation (e.g., ROS scavengers, PAD4 inhibitors, SYK or MAPK inhibitors) or enhance their clearance (e.g., DNase therapy, macrophage reprogramming) show promising results in preclinical models and early clinical studies, the inherent antimicrobial function of NETs necessitates caution to avoid compromising host defense. Therefore, future therapeutic approaches should prioritize precision and timing, aiming to modulate excessive or persistent NET activity without compromising their protective antimicrobial functions. Perhaps, strategies geared towards employing combination approaches that transiently dampen NET formation while simultaneously enhancing clearance may provide a more balanced and safer approach.

Current evidence increasingly indicates that severe viral infections are associated with dysregulated neutrophil function. However, the specific mechanisms, consequences, and therapeutic opportunities remain insufficiently defined, highlighting the need for targeted investigation and innovative approaches. Since many viruses engage distinct receptors or signaling cascades in neutrophils, a deeper understanding of virus-specific pathways for NET induction is essential for the development of tailored interventions for different viral diseases. Moreover, the exact molecular mechanisms governing neutrophil heterogeneity and reprogramming during viral infections remain incompletely understood. Therefore, the use of high-resolution approaches such as single-cell and spatial transcriptomics, combined with functional assays, could be pivotal in defining these pathogenic neutrophil subsets and elucidating their interactions with other immune and stromal cells.

In general, NETs should be viewed not as isolated entities but as integral components of a complex immunological network that involves other cell types, including platelets, macrophages, endothelial cells, and fibroblasts, all of which play key roles in disease pathology. Understanding the crosstalk between these cell types could reveal how the immune response shifts from protective immunity to pathological inflammation and tissue injury. This knowledge may help establish causal links between persistent NET signatures and acute or chronic viral syndromes, including hyperinflammation, fibrosis, vascular complications, and autoimmunity. Consequently, therapeutic approaches targeting this broader network, rather than neutrophils alone, may be more effective at preventing organ dysfunction and promoting durable recovery following severe viral infections.

In conclusion, NET release and NETosis sit at a pivotal crossroads of host–virus interactions, functioning as a rapid antimicrobial defense while also carrying substantial pathological potential. While NETs are indispensable for early antiviral defense, excessive or misdirected NET generation and defective clearance can drive a wide spectrum of pathological outcomes, including amplified inflammation, tissue injury, disrupted vascular and epithelial barriers, and the propagation of immunothrombotic and fibrotic sequelae. Specifically, knowledge that yields fine-tuned mechanistic and time-resolved insight into how NETs are initiated, shaped (including viable versus non-viable NETs), and ultimately cleared across tissues will be critical to preserve their protective functions while preventing collateral damage. As such, our improved understanding of neutrophil immunobiology will enable precision strategies to temper virus-driven hyperinflammation, reduce post-acute complications, and improve patient outcomes across diverse viral diseases.

## Figures and Tables

**Table 1:** Viral infections are associated with NET formation, impaired clearance, and NET-driven immunopathology.

Virus	Viral Family	Evidence of NET Formation	Effects on NET Clearance	Pathological Consequences Linked to NETs	References
SARS-CoV-2	Coronaviridae	Strong NET induction by spike protein, persistent NET-components in severe COVID-19 patients and immune complexes	Reduced DNase activity and macrophage clearance	Immunothrombosis, endothelial injury, lung fibrosis	[15,21,63]
Hepatitis B virus (HBV)	Hepadnaviridae	Induces NETs via S100A9-mediated neutrophil activation	Impaired neutrophil function and clearance	Liver fibrosis, cirrhosis, HCC progression	[7,25]
Influenza A virus	Orthomyxoviridae	Directly induces NET formation in neutrophils	Excess NET accumulation in lung tissue	Acute lung injury and ARDS	[10,64]
Respiratory syncytial virus (RSV)	Pneumoviridae	Viral fusion protein triggers NETosis via TLR signaling	NET persistence in airways	Airway obstruction and epithelial injury	[11,65]
Dengue virus	Flaviviridae	Strong NET induction via platelet-neutrophil interactions	Impaired degradation during severe disease	Vascular leakage and hemorrhage	[66]
HIV-1	Retroviridae	Induces NET release via TLR7/8 sensing of viral RNA	Viral factors reduce NET effectiveness	Chronic inflammation and immune activation	[69]
Chikungunya virus	Togaviridae	Strong NET induction through TLR7	Neutrophil activation and degranulation	Markers of inflammation and NET triggers	[67]
coxsackievirus B3- (CVB3)	Picornaviridae	NET induction through TLR8	NET persistence in cardiac tissues	Cardiac necrosis and inflammation	[68]

## Data Availability

No new data were generated for this review. All information discussed is derived from previously published studies and is presented within the article.

## Abbreviations

AAV, ANCA (anti-neutrophil cytoplasmic antibodies)-associated vasculitis; ACLF, acute-on-chronic liver failure; ADCC, antibody-dependent cellular cytotoxicity; ALI, acute lung injury; ARDS, acute respiratory distress syndrome; BAL, bronchoalveolar lavage; CitH3, citrullinated histone H3; DAG, diacylglycerol; DAMPs, damage-associated molecular patterns; DNase I, deoxyribonuclease I; eDNA, cell-free DNA; ECM, extracellular matrix; HBV, hepatitis B virus; HCC, hepatocellular carcinoma; HCV, hepatitis C virus; HDN, high-density neutrophil; HMGB1, high-mobility group box protein 1; IP3, inositol trisphosphate; LDN, low-density neutrophil; MAPK, mitogen-activated protein kinase; MMPs, matrix metalloproteinases; MOD, multi-organ dysfunction; MPO, myeloperoxidase; MS, multiple sclerosis; NAC, N-acetylcysteine; NE, neutrophil elastase; NET, neutrophil extracellular trap; NF-kB, Nuclear factor kappa-light-chain-enhancer of activated B cells; NOX, NADPH (nicotinamide adenine dinucleotide phosphate) oxidase; PAD4, peptidyl arginine deiminase type IV; PAMPs, pathogen-associated molecular patterns; PASC, post-acute sequelae of COVID-19; PKC, protein kinase C; PLCγ, phospholipase C gamma; PRR, pattern recognition receptor; PVT, portal vein thrombosis; RA, rheumatoid arthritis; ROS, reactive oxygen species; RSV, respiratory syncytial virus; SARS-CoV-2, severe acute respiratory syndrome coronavirus 2; scRNA-seq, single-cell RNA sequencing; SLE, systemic lupus erythematosus; SOD, superoxide dismutase; SYK, spleen tyrosine kinase; TLR, toll-like receptor; VWF, von Willebrand factor).

## References

[1] Vorobjeva N.V., Chernyak B.V. (2020). NETosis: Molecular Mechanisms, Role in Physiology and Pathology. Biochemistry.

[2] He Y., Yang F.Y., Sun E.W. (2018). Neutrophil Extracellular Traps in Autoimmune Diseases. Chin. Med. J..

[3] Krinsky N., Sizikov S., Nissim S., Dror A., Sas A., Prinz H., Pri-Or E., Perek S., Raz-Pasteur A., Lejbkowicz I. (2023). NETosis induction reflects COVID-19 severity and long COVID: Insights from a 2-center patient cohort study in Israel. J. Thromb. Haemost..

[4] Brinkmann V., Reichard U., Goosmann C., Fauler B., Uhlemann Y., Weiss D.S., Weinrauch Y., Zychlinsky A. (2004). Neutrophil extracellular traps kill bacteria. Science.

[5] Wang H., Kim S.J., Lei Y., Wang S., Wang H., Huang H., Zhang H., Tsung A. (2024). Neutrophil extracellular traps in homeostasis and disease. Signal Transduct. Target. Ther..

[6] Lebourgeois S., David A., Chenane H.R., Granger V., Menidjel R., Fidouh N., Noël B., Delelis O., Richetta C., Charpentier C. (2022). Differential activation of human neutrophils by SARS-CoV-2 variants of concern. Front. Immunol..

[7] Zhan X., Wu R., Kong X.H., You Y., He K., Sun X.Y., Huang Y., Chen W.X., Duan L. (2023). Elevated neutrophil extracellular traps by HBV-mediated S100A9-TLR4/RAGE-ROS cascade facilitate the growth and metastasis of hepatocellular carcinoma. Cancer Commun..

[8] Wei W.C., Liaw C.C., Tsai K.C., Chiou C.T., Tseng Y.H., Chiou W.F., Lin Y.C., Tsai C.I., Lin C.S., Lin C.S. (2022). Targeting spike protein-induced TLR/NET axis by COVID-19 therapeutic NRICM102 ameliorates pulmonary embolism and fibrosis. Pharmacol. Res..

[9] Youn Y.J., Lee Y.B., Kim S.H., Jin H.K., Bae J.S., Hong C.W. (2021). Nucleocapsid and Spike Proteins of SARS-CoV-2 Drive Neutrophil Extracellular Trap Formation. Immune Netw..

[10] Chan L.L.Y., Nicholls J.M., Peiris J.S.M., Lau Y.L., Chan M.C.W., Chan R.W.Y. (2020). Host DNA released by NETosis in neutrophils exposed to seasonal H1N1 and highly pathogenic H5N1 influenza viruses. Respir. Res..

[11] Muraro S.P., De Souza G.F., Gallo S.W., Da Silva B.K., De Oliveira S.D., Vinolo M.A.R., Saraiva E.M., Porto B.N. (2018). Respiratory Syncytial Virus induces the classical ROS-dependent NETosis through PAD-4 and necroptosis pathways activation. Sci. Rep..

[12] Backman E., Gröning R., Lind A., Granvik C., Eilers H., Lange A., Ahlm C., Cajander S., Forsell M.N.E., Normark J. (2025). Elevated Plasma Levels of NET Components in Men with Severe COVID-19 Correlates to Increased Amounts of IL-18. Eur. J. Immunol..

[13] Tanaka A., Wakayama K., Fukuda Y., Ohta S., Homma T., Ando K., Nishihara Y., Nakano R., Zhao J., Suzuki Y. (2024). Increased levels of circulating cell-free DNA in COVID-19 patients with respiratory failure. Sci. Rep..

[14] Mahmoodpoor A., Mohammadzadeh M., Asghari R., Tagizadeh M., Iranpour A., Rezayi M., Pahnvar A.J., Emamalizadeh B., Sohrabifar N., Kazeminasab S. (2023). Prognostic potential of circulating cell free mitochondrial DNA levels in COVID-19 patients. Mol. Biol. Rep..

[15] Asaba C.N., Bitazar R., Labonté P., Bukong T.N. (2025). Bronchoalveolar lavage single-cell transcriptomics reveals immune dysregulations driving COVID-19 severity. PLoS ONE.

[16] Wang Y., Zhang C., Wang H., Zhou Y., Yu Y., Liu H., Gu C. (2026). Research progress on neutrophil extracellular traps and hepatitis-to-hepatocellular carcinoma transformation (Review). Oncol. Lett..

[17] Chen J., He R., Luo J., Yan S., Zhu W., Liu S. (2025). Neutrophil Extracellular Traps in Viral Infections. Pathogens.

[18] Monsalve D.M., Acosta-Ampudia Y., Acosta N.G., Celis-Andrade M., Şahin A., Yilmaz A.M., Shoenfeld Y., Ramírez-Santana C. (2025). NETosis: A key player in autoimmunity, COVID-19, and long COVID. J. Transl. Autoimmun..

[19] Heneghan M.A., Lohse A.W. (2025). Update in clinical science: Autoimmune hepatitis. J. Hepatol..

[20] Domerecka W., Homa-Mlak I., Mlak R., Michalak A., Wilińska A., Kowalska-Kępczyńska A., Dreher P., Cichoż-Lach H., Małecka-Massalska T. (2022). Indicator of Inflammation and NETosis-Low-Density Granulocytes as a Biomarker of Autoimmune Hepatitis. J. Clin. Med..

[21] Morrissey S.M., Geller A.E., Hu X., Tieri D., Ding C., Klaes C.K., Cooke E.A., Woeste M.R., Martin Z.C., Chen O. (2021). A specific low-density neutrophil population correlates with hypercoagulation and disease severity in hospitalized COVID-19 patients. JCI Insight.

[22] Dwivedi A., Ui Mhaonaigh A., Carroll M., Khosravi B., Batten I., Ballantine R.S., Hendricken Phelan S., O’Doherty L., George A.M., Sui J. (2024). Emergence of dysfunctional neutrophils with a defect in arginase-1 release in severe COVID-19. JCI Insight.

[23] Tay S.H., Celhar T., Fairhurst A.M. (2020). Low-Density Neutrophils in Systemic Lupus Erythematosus. Arthritis Rheumatol..

[24] Bukong T.N., Cho Y., Iracheta-Vellve A., Saha B., Lowe P., Adejumo A., Furi I., Ambade A., Gyongyosi B., Catalano D. (2018). Abnormal neutrophil traps and impaired efferocytosis contribute to liver injury and sepsis severity after binge alcohol use. J. Hepatol..

[25] Wu W., Sun S., Wang Y., Zhao R., Ren H., Li Z., Zhao H., Zhang Y., Sheng J., Chen Z. (2021). Circulating Neutrophil Dysfunction in HBV-Related Acute-on-Chronic Liver Failure. Front. Immunol..

[26] Xue J., Dong P., Pida M., Ma K., Zhao H., Chen J., Wu X. (2025). Evaluation of peripheral blood neutrophil extracellular traps as fibrotic markers in patients with chronic hepatitis B. Arab. J. Gastroenterol..

[27] Dhawan U.K., Vartak T., Englert H., Russo S., Vasconcellos L.R.C., Singhal A., Chakraborty R., Bhagat K.K., McDonnell C., Connolly M. (2025). Macrophage DNases Limit Neutrophil Extracellular Trap-Mediated Defective Efferocytosis in Atherosclerosis. Circ. Res..

[28] Shahzad A., Ni Y., Yang Y., Liu W., Teng Z., Bai H., Liu X., Sun Y., Xia J., Cui K. (2025). Neutrophil Extracellular Traps (NETs) in health and disease. Mol. Biomed..

[29] Yu S., Liu J., Yan N. (2022). Endothelial Dysfunction Induced by Extracellular Neutrophil Traps Plays Important Role in the Occurrence and Treatment of Extracellular Neutrophil Traps-Related Disease. Int. J. Mol. Sci..

[30] Li X., Ye Y., Peng K., Zeng Z., Chen L., Zeng Y. (2022). Histones: The critical players in innate immunity. Front. Immunol..

[31] Apel F., Andreeva L., Knackstedt L.S., Streeck R., Frese C.K., Goosmann C., Hopfner K.P., Zychlinsky A. (2021). The cytosolic DNA sensor cGAS recognizes neutrophil extracellular traps. Sci. Signal.

[32] Hong W., Yang J., Zou J., Bi Z., He C., Lei H., He X., Li X., Alu A., Ren W. (2022). Histones released by NETosis enhance the infectivity of SARS-CoV-2 by bridging the spike protein subunit 2 and sialic acid on host cells. Cell Mol. Immunol..

[33] Xia Y., Wang Y., Xiong Q., He J., Wang H., Islam M., Zhou X., Kim A., Zhang H., Huang H. (2025). Neutrophil extracellular traps promote MASH fibrosis by metabolic reprogramming of HSC. Hepatology.

[34] Shen J., Huang S., Wang Y., Wang Q., Lin S., Guan W., Gong Y., Si Y., Zhao M., Zhou H. (2026). PAD4+ neutrophils promote hepatic stellate cell activation and accelerate MASH fibrosis progression viaNET-DNA/TAOK1/MAPK pathways. JCI Insight.

[35] Mak K.M., Shekhar A.C., Ding S.Y. (2026). Neutrophil extracellular traps mediate pathophysiology of hepatic cells during liver injury. Anat. Rec..

[36] Deinhardt-Emmer S., Böttcher S., Häring C., Giebeler L., Henke A., Zell R., Jungwirth J., Jordan P.M., Werz O., Hornung F. (2021). SARS-CoV-2 causes severe epithelial inflammation and barrier dysfunction. J. Virol..

[37] Cai J., Ma W., Wang X., Chang H., Wei Z., Li J., Zeng M. (2023). The spike protein of SARS-CoV-2 induces inflammation and EMT of lung epithelial cells and fibroblasts through the upregulation of GADD45A. Open Med..

[38] Chen H., Liu W., Wang Y., Liu D., Zhao L., Yu J. (2021). SARS-CoV-2 activates lung epithelial cell proinflammatory signaling and leads to immune dysregulation in COVID-19 patients. eBioMedicine.

[39] Park J.H., Lee H.K. (2020). Re-analysis of Single Cell Transcriptome Reveals That the NR3C1-CXCL8-Neutrophil Axis Determines the Severity of COVID-19. Front. Immunol..

[40] Melero I., Villalba-Esparza M., Recalde-Zamacona B., Jiménez-Sánchez D., Teijeira Á., Argueta A., García-Tobar L., Álvarez-Gigli L., Sainz C., Garcia-Ros D. (2022). Neutrophil Extracellular Traps, Local IL-8 Expression, and Cytotoxic T-Lymphocyte Response in the Lungs of Patients with Fatal COVID-19. Chest.

[41] Gehring A.J., Koh S., Chia A., Paramasivam K., Chew V.S., Ho Z.Z., Lee K.H., Maini M.K., Madhavan K., Lim S.G. (2011). Licensing virus-specific T cells to secrete the neutrophil attracting chemokine CXCL-8 during hepatitis B virus infection. PLoS ONE.

[42] Iwaniuk A., Jablonska E. (2023). Neutrophils in Health and Disease: From Receptor Sensing to Inflammasome Activation. Int. J. Mol. Sci..

[43] Stegelmeier A.A., Darzianiazizi M., Hanada K., Sharif S., Wootton S.K., Bridle B.W., Karimi K. (2021). Type I Interferon-Mediated Regulation of Antiviral Capabilities of Neutrophils. Int. J. Mol. Sci..

[44] Funchal G.A., Jaeger N., Czepielewski R.S., Machado M.S., Muraro S.P., Stein R.T., Bonorino C.B., Porto B.N. (2015). Respiratory syncytial virus fusion protein promotes TLR-4-dependent neutrophil extracellular trap formation by human neutrophils. PLoS ONE.

[45] Sung P.S., Yang S.P., Peng Y.C., Sun C.P., Tao M.H., Hsieh S.L. (2022). CLEC5A and TLR2 are critical in SARS-CoV-2-induced NET formation and lung inflammation. J. Biomed. Sci..

[46] Shahrakyvahed A., Sanchooli J., Sanadgol N., Arababadi M.K., Kennedy D. (2014). TLR9: An important molecule in the fight against hepatitis B virus. Postgrad. Med. J..

[47] Rehwinkel J., Gack M.U. (2020). RIG-I-like receptors: Their regulation and roles in RNA sensing. Nat. Rev. Immunol..

[48] Vieira S.M., Lemos H.P., Grespan R., Napimoga M.H., Dal-Secco D., Freitas A., Cunha T.M., Verri W.A., Souza-Junior D.A., Jamur M.C. (2009). A crucial role for TNF-alpha in mediating neutrophil influx induced by endogenously generated or exogenous chemokines, KC/CXCL1 and LIX/CXCL5. Br. J. Pharmacol..

[49] Worley M.J., Fei K., Lopez-Denman A.J., Kelleher A.D., Kent S.J., Chung A.W. (2018). Neutrophils mediate HIV-specific antibody-dependent phagocytosis and ADCC. J. Immunol. Methods.

[50] Allen K.C., Warner S., Teague H.L., Ramos-Benitez M.J., Miao R., Tian X., Reger R., Burbelo P.D., Pang C.W.J., Kanthi Y. (2025). SARS-CoV-2 Immune Complex-Mediated Neutrophil Activation. Open Forum Infect. Dis..

[51] Zhang A., Stacey H.D., D’Agostino M.R., Tugg Y., Marzok A., Miller M.S. (2023). Beyond neutralization: Fc-dependent antibody effector functions in SARS-CoV-2 infection. Nat. Rev. Immunol..

[52] Strich J.R., Ramos-Benitez M.J., Randazzo D., Stein S.R., Babyak A., Davey R.T., Suffredini A.F., Childs R.W., Chertow D.S. (2021). Fostamatinib Inhibits Neutrophils Extracellular Traps Induced by COVID-19 Patient Plasma: A Potential Therapeutic. J. Infect. Dis..

[53] Zhang S., Wang L., Lu Y., Guo C., Zhang T., Zhang L. (2025). Targeting spleen tyrosine kinase (SYK): Structure, mechanisms and drug discovery. Drug Discov. Today.

[54] Chen T., Li Y., Sun R., Hu H., Liu Y., Herrmann M., Zhao Y., Muñoz L.E. (2021). Receptor-Mediated NETosis on Neutrophils. Front. Immunol..

[55] Tatsiy O., McDonald P.P. (2018). Physiological Stimuli Induce PAD4-Dependent, ROS-Independent NETosis, with Early and Late Events Controlled by Discrete Signaling Pathways. Front. Immunol..

[56] Li Y., Li M., Weigel B., Mall M., Werth V.P., Liu M.L. (2020). Nuclear envelope rupture and NET formation is driven by PKCα-mediated lamin B disassembly. EMBO Rep..

[57] Chen K.W., Monteleone M., Boucher D., Sollberger G., Ramnath D., Condon N.D., von Pein J.B., Broz P., Sweet M.J., Schroder K. (2018). Noncanonical inflammasome signaling elicits gasdermin D-dependent neutrophil extracellular traps. Sci. Immunol..

[58] Hu S., Liu X., Gao Y., Zhou R., Wei M., Dong J., Yan H., Zhao Y. (2019). Hepatitis B Virus Inhibits Neutrophil Extracellular Trap Release by Modulating Reactive Oxygen Species Production and Autophagy. J. Immunol..

[59] Serrano-Gonzalo I., Menéndez-Jandula B., Franco-García E., Arévalo-Vargas I., Lahoz-Gil C., Latre P., Roca-Esteve S., Köhler R., López de Frutos L., Giraldo P. (2025). Neutrophil extracellular traps and macrophage activation contibute to thrombosis and post-covid syndrome in SARS-CoV-2 infection. Front. Immunol..

[60] Cho Y., Bukong T.N., Tornai D., Babuta M., Vlachos I.S., Kanata E., Catalano D., Szabo G. (2023). Neutrophil extracellular traps contribute to liver damage and increase defective low-density neutrophils in alcohol-associated hepatitis. J. Hepatol..

[61] Eddins D.J., Yang J., Kosters A., Giacalone V.D., Pechuan-Jorge X., Chandler J.D., Eum J., Babcock B.R., Dobosh B.S., Hernández M.R. (2023). Transcriptional reprogramming of infiltrating neutrophils drives lung pathology in severe COVID-19 despite low viral load. Blood Adv..

[62] Creusat F., Jouan Y., Gonzalez L., Barsac E., Ilango G., Lemoine R., Soulard D., Hankard A., Boisseau C., Guillon A. (2024). IFN-γ primes bone marrow neutrophils to acquire regulatory functions in severe viral respiratory infections. Sci. Adv..

[63] Belchamber K.B.R., Thein O.S., Hazeldine J., Grudzinska F.S., Faniyi A.A., Hughes M.J., Jasper A.E., Yip K.P., Crowley L.E., Lugg S.T. (2022). Dysregulated Neutrophil Phenotype and Function in Hospitalised Non-ICU COVID-19 Pneumonia. Cells.

[64] Rudd J.M., Pulavendran S., Ashar H.K., Ritchey J.W., Snider T.A., Malayer J.R., Marie M., Chow V.T.K., Narasaraju T. (2019). Neutrophils Induce a Novel Chemokine Receptors Repertoire During Influenza Pneumonia. Front. Cell. Infect. Microbiol..

[65] Cortjens B., de Boer O.J., de Jong R., Antonis A.F., Sabogal Piñeros Y.S., Lutter R., van Woensel J.B., Bem R.A. (2016). Neutrophil extracellular traps cause airway obstruction during respiratory syncytial virus disease. J. Pathol..

[66] Opasawatchai A., Amornsupawat P., Jiravejchakul N., Chan-In W., Spoerk N.J., Manopwisedjaroen K., Singhasivanon P., Yingtaweesak T., Suraamornkul S., Mongkolsapaya J. (2018). Neutrophil Activation and Early Features of NET Formation Are Associated with Dengue Virus Infection in Human. Front. Immunol..

[67] de Souza W.M., Fumagalli M.J., de Lima S.T.S., Parise P.L., Carvalho D.C.M., Hernandez C., de Jesus R., Delafiori J., Candido D.S., Carregari V.C. (2024). Pathophysiology of chikungunya virus infection associated with fatal outcomes. Cell Host Microbe.

[68] Carai P., González L.F., Van Bruggen S., Spalart V., De Giorgio D., Geuens N., Martinod K., Jones E.A.V., Heymans S. (2023). Neutrophil inhibition improves acute inflammation in a murine model of viral myocarditis. Cardiovasc. Res..

[69] Saitoh T., Komano J., Saitoh Y., Misawa T., Takahama M., Kozaki T., Uehata T., Iwasaki H., Omori H., Yamaoka S. (2012). Neutrophil extracellular traps mediate a host defense response to human immunodeficiency virus-1. Cell Host Microbe.

[70] Aubé F.A., Bidias A., Pépin G. (2023). Who and how, DNA sensors in NETs-driven inflammation. Front. Immunol..

[71] Zhou Z., Ou-Yang C., Chen Q., Ren Z., Guo X., Lei M., Liu C., Yang X. (2023). Trafficking and effect of released DNA on cGAS-STING signaling pathway and cardiovascular disease. Front. Immunol..

[72] Blanco L.P., Wang X., Carlucci P.M., Torres-Ruiz J.J., Romo-Tena J., Sun H.W., Hafner M., Kaplan M.J. (2021). RNA Externalized by Neutrophil Extracellular Traps Promotes Inflammatory Pathways in Endothelial Cells. Arthritis Rheumatol..

[73] Tsourouktsoglou T.D., Warnatsch A., Ioannou M., Hoving D., Wang Q., Papayannopoulos V. (2020). Histones, DNA, and Citrullination Promote Neutrophil Extracellular Trap Inflammation by Regulating the Localization and Activation of TLR4. Cell Rep..

[74] Wang X., Mayorga-Flores M., Bien K.G., Bailey A.O., Iwahara J. (2022). DNA-mediated proteolysis by neutrophil elastase enhances binding activities of the HMGB1 protein. J. Biol. Chem..

[75] Szturmowicz M., Demkow U. (2021). Neutrophil Extracellular Traps (NETs) in Severe SARS-CoV-2 Lung Disease. Int. J. Mol. Sci..

[76] Giryes S., Bragazzi N.L., Bridgewood C., De Marco G., McGonagle D. (2022). COVID-19 Vasculitis and vasculopathy-Distinct immunopathology emerging from the close juxtaposition of Type II Pneumocytes and Pulmonary Endothelial Cells. Semin. Immunopathol..

[77] Ackermann M., Anders H.J., Bilyy R., Bowlin G.L., Daniel C., De Lorenzo R., Egeblad M., Henneck T., Hidalgo A., Hoffmann M. (2021). Patients with COVID-19: In the dark-NETs of neutrophils. Cell Death Differ..

[78] Caillon A., Trimaille A., Favre J., Jesel L., Morel O., Kauffenstein G. (2022). Role of neutrophils, platelets, and extracellular vesicles and their interactions in COVID-19-associated thrombopathy. J. Thromb. Haemost..

[79] Hirsch J., Uzun G., Zlamal J., Singh A., Bakchoul T. (2023). Platelet-neutrophil interaction in COVID-19 and vaccine-induced thrombotic thrombocytopenia. Front. Immunol..

[80] Klenk C., Erber J., Fresacher D., Röhrl S., Lengl M., Heim D., Irl H., Schlegel M., Haller B., Lahmer T. (2023). Platelet aggregates detected using quantitative phase imaging associate with COVID-19 severity. Commun. Med..

[81] Hottz E.D., Bozza P.T. (2022). Platelet-leukocyte interactions in COVID-19: Contributions to hypercoagulability, inflammation, and disease severity. Res. Pract. Thromb. Haemost..

[82] Lopez-Castaneda S., García-Larragoiti N., Cano-Mendez A., Blancas-Ayala K., Damian-Vázquez G., Perez-Medina A.I., Chora-Hernández L.D., Arean-Martínez C., Viveros-Sandoval M.E. (2021). Inflammatory and Prothrombotic Biomarkers Associated with the Severity of COVID-19 Infection. Clin. Appl. Thromb. Hemost..

[83] Obermayer A., Jakob L.M., Haslbauer J.D., Matter M.S., Tzankov A., Stoiber W. (2021). Neutrophil Extracellular Traps in Fatal COVID-19-Associated Lung Injury. Dis. Markers.

[84] Middleton E.A., He X.Y., Denorme F., Campbell R.A., Ng D., Salvatore S.P., Mostyka M., Baxter-Stoltzfus A., Borczuk A.C., Loda M. (2020). Neutrophil extracellular traps contribute to immunothrombosis in COVID-19 acute respiratory distress syndrome. Blood.

[85] Li Z., Jiang Y., Wang S., Qiao Y., Kong X., Jing X. (2025). Correlation between virological response and portal vein thrombosis in patients with chronic hepatitis B. Sci. Rep..

[86] Lertpipopmetha K., Auewarakul C.U. (2011). High incidence of hepatitis B infection-associated cirrhosis and hepatocellular carcinoma in the Southeast Asian patients with portal vein thrombosis. BMC Gastroenterol..

[87] Han M., Liu Y., Cao Y., Zhang Y., Yan Y., Deng S., Yuan X., Xing H., Huang Y., Zhu L. (2024). The Imbalance of Homeostasis in Neutrophil Extracellular Traps is Associated with Portal Vein Thrombosis in Patients with Decompensated Cirrhosis. J. Clin. Transl. Hepatol..

[88] Muhammad I., Contes K., Bility M.T., Tang Q. (2025). Chasing Virus Replication and Infection: PAMP-PRR Interaction Drives Type I Interferon Production, Which in Turn Activates ISG Expression and ISGylation. Viruses.

[89] Asaba C.N., Ekabe C.J., Ayuk H.S., Gwanyama B.N., Bitazar R., Bukong T.N. (2024). Interplay of TLR4 and SARS-CoV-2: Unveiling the Complex Mechanisms of Inflammation and Severity in COVID-19 Infections. J. Inflamm. Res..

[90] Lupfer C., Malik A., Kanneganti T.D. (2015). Inflammasome control of viral infection. Curr. Opin. Virol..

[91] Narasaraju T., Tang B.M., Herrmann M., Muller S., Chow V.T.K., Radic M. (2020). Neutrophilia and NETopathy as Key Pathologic Drivers of Progressive Lung Impairment in Patients with COVID-19. Front. Pharmacol..

[92] Li J., Shan R., Miller H., Filatov A., Byazrova M.G., Yang L., Liu C. (2025). The roles of macrophages and monocytes in COVID-19 Severe Respiratory Syndrome. Cell Insight.

[93] Khalil B.A., Elemam N.M., Maghazachi A.A. (2021). Chemokines and chemokine receptors during COVID-19 infection. Comput. Struct. Biotechnol. J..

[94] Zuo Y., Yalavarthi S., Shi H., Gockman K., Zuo M., Madison J.A., Blair C., Weber A., Barnes B.J., Egeblad M. (2020). Neutrophil extracellular traps in COVID-19. JCI Insight.

[95] Veras F.P., Pontelli M.C., Silva C.M., Toller-Kawahisa J.E., de Lima M., Nascimento D.C., Schneider A.H., Caetité D., Tavares L.A., Paiva I.M. (2020). SARS-CoV-2-triggered neutrophil extracellular traps mediate COVID-19 pathology. J. Exp. Med..

[96] Wynn T.A., Vannella K.M. (2016). Macrophages in Tissue Repair, Regeneration, and Fibrosis. Immunity.

[97] Radermecker C., Detrembleur N., Guiot J., Cavalier E., Henket M., d’Emal C., Vanwinge C., Cataldo D., Oury C., Delvenne P. (2020). Neutrophil extracellular traps infiltrate the lung airway, interstitial, and vascular compartments in severe COVID-19. J. Exp. Med..

[98] Battaglia D.M., Post C.E., Yao W., Wahl A., Gralinski L.E., Liu H., Dang H., Madden V.J., White K.K., Leist S.R. (2025). SARS-CoV-2 infection induces pro-fibrotic and pro-thrombotic foam cell formation. Nat. Microbiol..

[99] Esendağli D., Yilmaz A., Akçay Ş., Özlü T. (2021). Post-COVID syndrome: Pulmonary complications. Turk. J. Med. Sci..

[100] Andersson U., Ottestad W., Tracey K.J. (2020). Extracellular HMGB1: A therapeutic target in severe pulmonary inflammation including COVID-19?. Mol. Med..

[101] Wang Q., Wang J., Wang J., Hong S., Han F., Chen J., Chen G. (2017). HMGB1 induces lung fibroblast to myofibroblast differentiation through NF-κB-mediated TGF-β1 release. Mol. Med. Rep..

[102] Torres-Ruiz J., Absalón-Aguilar A., Nuñez-Aguirre M., Pérez-Fragoso A., Carrillo-Vázquez D.A., Maravillas-Montero J.L., Mejía-Domínguez N.R., Llorente L., Alcalá-Carmona B., Lira-Luna J. (2021). Neutrophil Extracellular Traps Contribute to COVID-19 Hyperinflammation and Humoral Autoimmunity. Cells.

[103] Yan S., Li M., Liu B., Ma Z., Yang Q. (2023). Neutrophil extracellular traps and pulmonary fibrosis: An update. J. Inflamm..

[104] Zheng Z., Peng F., Zhou Y. (2023). Pulmonary fibrosis: A short- or long-term sequelae of severe COVID-19?. Chin. Med. J. Pulm. Crit. Care Med..

[105] Kamiya M., Carter H., Espindola M.S., Doyle T.J., Lee J.S., Merriam L.T., Zhang F., Kawano-Dourado L., Sparks J.A., Hogaboam C.M. (2024). Immune mechanisms in fibrotic interstitial lung disease. Cell.

[106] Gustine J.N., Jones D. (2021). Immunopathology of Hyperinflammation in COVID-19. Am. J. Pathol..

[107] MacDonald L., Alivernini S., Tolusso B., Elmesmari A., Somma D., Perniola S., Paglionico A., Petricca L., Bosello S.L., Carfì A. (2021). COVID-19 and RA share an SPP1 myeloid pathway that drives PD-L1+ neutrophils and CD14+ monocytes. JCI Insight.

[108] Bility M.T., Cheng L., Zhang Z., Luan Y., Li F., Chi L., Zhang L., Tu Z., Gao Y., Fu Y. (2014). Hepatitis B virus infection and immunopathogenesis in a humanized mouse model: Induction of human-specific liver fibrosis and M2-like macrophages. PLoS Pathog..

[109] Ding L., Guo H., Zhang C., Jin H., Guo X., Li T. (2023). Elevated matrix metalloproteinase-9 expression is associated with COVID-19 severity: A meta-analysis. Exp. Ther. Med..

[110] Castanheira F.V.S., Kubes P. (2023). Neutrophils during SARS-CoV-2 infection: Friend or foe?. Immunol. Rev..

[111] Fajgenbaum D.C., June C.H. (2020). Cytokine Storm. N. Engl. J. Med..

[112] Rodrigues P.R.S., Alrubayyi A., Pring E., Bart V.M.T., Jones R., Coveney C., Lu F., Tellier M., Maleki-Toyserkani S., Richter F.C. (2020). Innate immunology in COVID-19-a living review. Part II: Dysregulated inflammation drives immunopathology. Oxf. Open Immunol..

[113] Shaath H., Vishnubalaji R., Elkord E., Alajez N.M. (2020). Single-Cell Transcriptome Analysis Highlights a Role for Neutrophils and Inflammatory Macrophages in the Pathogenesis of Severe COVID-19. Cells.

[114] Cheung C.C.L., Mok C.C. (2025). Long COVID in patients with systemic lupus erythematosus: A case-control study. Lupus.

[115] Yadav S., Bonnes S.L., Gilman E.A., Mueller M.R., Collins N.M., Hurt R.T., Ganesh R. (2023). Inflammatory Arthritis After COVID-19: A Case Series. Am. J. Case Rep..

[116] Fernandes de Souza W.D., Fonseca D.M.D., Sartori A. (2023). COVID-19 and Multiple Sclerosis: A Complex Relationship Possibly Aggravated by Low Vitamin D Levels. Cells.

[117] Du S.N., Chen J.W., Li W., Wang M.C., Mao Y.S. (2024). Development of autoimmune thyroid disease after COVID-19 infection: Case report. Front. Med..

[118] Heald A.H., Williams R., Jenkins D.A., Stewart S., Bakerly N.D., McCay K., Ollier W. (2024). The prevalence of long COVID in people with diabetes mellitus-evidence from a UK cohort. eClinicalMedicine.

[119] Rojas M., Rodríguez Y., Acosta-Ampudia Y., Monsalve D.M., Zhu C., Li Q.Z., Ramírez-Santana C., Anaya J.M. (2022). Autoimmunity is a hallmark of post-COVID syndrome. J. Transl. Med..

[120] Sadeghi M., Dehnavi S., Jamialahmadi T., Johnston T.P., Sahebkar A. (2023). Neutrophil extracellular trap: A key player in the pathogenesis of autoimmune diseases. Int. Immunopharmacol..

[121] Hattori F., Nishiyama J., Hasuo H. (2025). Correlation of interferons and autoimmune aspects in long COVID-19 patients. Int. Immunol..

[122] Lee J.S., Park S., Jeong H.W., Ahn J.Y., Choi S.J., Lee H., Choi B., Nam S.K., Sa M., Kwon J.S. (2020). Immunophenotyping of COVID-19 and influenza highlights the role of type I interferons in development of severe COVID-19. Sci. Immunol..

[123] Lucas C., Wong P., Klein J., Castro T.B.R., Silva J., Sundaram M., Ellingson M.K., Mao T., Oh J.E., Israelow B. (2020). Longitudinal analyses reveal immunological misfiring in severe COVID-19. Nature.

[124] Spengler J., Lugonja B., Ytterberg A.J., Zubarev R.A., Creese A.J., Pearson M.J., Grant M.M., Milward M., Lundberg K., Buckley C.D. (2015). Release of Active Peptidyl Arginine Deiminases by Neutrophils Can Explain Production of Extracellular Citrullinated Autoantigens in Rheumatoid Arthritis Synovial Fluid. Arthritis Rheumatol..

[125] Gregorio G.V., Jones H., Choudhuri K., Vegnente A., Bortolotti F., Mieli-Vergani G., Vergani D. (1996). Autoantibody prevalence in chronic hepatitis B virus infection: Effect in interferon alfa. Hepatology.

[126] Mazzaro C., Adinolfi L.E., Pozzato G., Nevola R., Zanier A., Serraino D., Andreone P., Fenoglio R., Sciascia S., Gattei V. (2022). Extrahepatic Manifestations of Chronic HBV Infection and the Role of Antiviral Therapy. J. Clin. Med..

[127] Maya R., Gershwin M.E., Shoenfeld Y. (2008). Hepatitis B virus (HBV) and autoimmune disease. Clin. Rev. Allergy Immunol..

[128] Bhagoowani S., Devi U., Munir A., Hasnain U., Iqbal J. (2024). Antineutrophil cytoplasmic antibodies (ANCA)-associated vasculitis in chronic Hepatitis B: Unraveling the immune puzzle—A rare case report with review of literature. IDCases.

[129] Rohrbach A.S., Slade D.J., Thompson P.R., Mowen K.A. (2012). Activation of PAD4 in NET formation. Front. Immunol..

[130] Liu X., Arfman T., Wichapong K., Reutelingsperger C.P.M., Voorberg J., Nicolaes G.A.F. (2021). PAD4 takes charge during neutrophil activation: Impact of PAD4 mediated NET formation on immune-mediated disease. J. Thromb. Haemost..

[131] Lewis H.D., Liddle J., Coote J.E., Atkinson S.J., Barker M.D., Bax B.D., Bicker K.L., Bingham R.P., Campbell M., Chen Y.H. (2015). Inhibition of PAD4 activity is sufficient to disrupt mouse and human NET formation. Nat. Chem. Biol..

[132] Katsumata M., Ikari J., Urano A., Suzuki E., Kugou K., Hasegawa Y., Tatsumi K., Suzuki T. (2025). Peptidylarginine Deiminase 4 Deficiency Suppresses Neutrophil Extracellular Trap Formation and Ameliorates Elastase-Induced Emphysema in Mouse Lung. Int. J. Mol. Sci..

[133] Suzuki M., Ikari J., Anazawa R., Tanaka N., Katsumata Y., Shimada A., Suzuki E., Tatsumi K. (2020). PAD4 Deficiency Improves Bleomycin-induced Neutrophil Extracellular Traps and Fibrosis in Mouse Lung. Am. J. Respir. Cell Mol. Biol..

[134] Kijak-Boćkowska M., Czerwińska J., Owczarczyk-Saczonek A. (2025). Peptidylarginine Deiminases: An Overview of Recent Advances in Citrullination Research. Int. J. Mol. Sci..

[135] Knight J.S., Subramanian V., O’Dell A.A., Yalavarthi S., Zhao W., Smith C.K., Hodgin J.B., Thompson P.R., Kaplan M.J. (2015). Peptidylarginine deiminase inhibition disrupts NET formation and protects against kidney, skin and vascular disease in lupus-prone MRL/lpr mice. Ann. Rheum. Dis..

[136] Zhu D., Lu Y., Wang Y., Wang Y. (2022). PAD4 and Its Inhibitors in Cancer Progression and Prognosis. Pharmaceutics.

[137] Jia Y., Jia R., Taledaohan A., Wang Y., Wang Y. (2024). Structure-Activity Relationship of PAD4 Inhibitors and Their Role in Tumor Immunotherapy. Pharmaceutics.

[138] Urban C.F., Reichard U., Brinkmann V., Zychlinsky A. (2006). Neutrophil extracellular traps capture and kill *Candida albicans* yeast and hyphal forms. Cell Microbiol..

[139] Abi Abdallah D.S., Lin C., Ball C.J., King M.R., Duhamel G.E., Denkers E.Y. (2012). Toxoplasma gondii triggers release of human and mouse neutrophil extracellular traps. Infect. Immun..

[140] Lopes B.R.P., da Silva G.S., de Lima Menezes G., de Oliveira J., Watanabe A.S.A., Porto B.N., da Silva R.A., Toledo K.A. (2022). Serine proteases in neutrophil extracellular traps exhibit anti-Respiratory Syncytial Virus activity. Int. Immunopharmacol..

[141] Meng W., Paunel-Görgülü A., Flohé S., Hoffmann A., Witte I., MacKenzie C., Baldus S.E., Windolf J., Lögters T.T. (2012). Depletion of neutrophil extracellular traps in vivo results in hypersusceptibility to polymicrobial sepsis in mice. Crit. Care.

[142] King P.T., Dousha L. (2024). Neutrophil Extracellular Traps and Respiratory Disease. J. Clin. Med..

[143] Manoharan R.R., Zachová K., Buzáš M., Pospíšil P., Křupka M., Prasad A. (2024). NADPH oxidase-dependent free radical generation and protein adduct formation in neutrophils. RSC Adv..

[144] Azzouz D., Palaniyar N. (2024). How Do ROS Induce NETosis? Oxidative DNA Damage, DNA Repair, and Chromatin Decondensation. Biomolecules.

[145] Kalyanaraman B. (2022). NAC, NAC, Knockin’ on Heaven’s door: Interpreting the mechanism of action of N-acetylcysteine in tumor and immune cells. Redox Biol..

[146] Tenório M., Graciliano N.G., Moura F.A., Oliveira A.C.M., Goulart M.O.F. (2021). N-Acetylcysteine (NAC): Impacts on Human Health. Antioxidants.

[147] Aldini G., Altomare A., Baron G., Vistoli G., Carini M., Borsani L., Sergio F. (2018). N-Acetylcysteine as an antioxidant and disulphide breaking agent: The reasons why. Free Radic. Res..

[148] Metzler K.D., Goosmann C., Lubojemska A., Zychlinsky A., Papayannopoulos V. (2014). A myeloperoxidase-containing complex regulates neutrophil elastase release and actin dynamics during NETosis. Cell Rep..

[149] He T., Ren K., Xiang L., Yao H., Huang Y., Gao Y. (2024). Efficacy of N-Acetylcysteine as an Adjuvant Therapy for Rheumatoid Arthritis: A Systematic Review and Meta-Analysis of Randomized Controlled Trials. Br. J. Hosp. Med..

[150] Di Paola R., Mazzon E., Zito D., Maiere D., Britti D., Genovese T., Cuzzocrea S. (2005). Effects of Tempol, a membrane-permeable radical scavenger, in a rodent model periodontitis. J. Clin. Periodontol..

[151] Wilcox C.S. (2010). Effects of tempol and redox-cycling nitroxides in models of oxidative stress. Pharmacol. Ther..

[152] Reddan J., Sevilla M., Giblin F., Padgaonkar V., Dziedzic D., Leverenz V. (1992). Tempol and deferoxamine protect cultured rabbit lens epithelial cells from H_2_O_2_ insult: Insight into the mechanism of H_2_O_2_-induced injury. Lens Eye Toxic. Res..

[153] Laight D.W., Andrews T.J., Haj-Yehia A.I., Carrier M.J., Anggård E.E. (1997). Microassay of superoxide anion scavenging activity in vitro. Environ. Toxicol. Pharmacol..

[154] Abouzied M.M., Eltahir H.M., Taye A., Abdelrahman M.S. (2016). Experimental evidence for the therapeutic potential of tempol in the treatment of acute liver injury. Mol. Cell. Biochem..

[155] Toyoda K., Fujii K., Kamouchi M., Nakane H., Arihiro S., Okada Y., Ibayashi S., Iida M. (2004). Free radical scavenger, edaravone, in stroke with internal carotid artery occlusion. J. Neurol. Sci..

[156] Cha S.J., Kim K. (2022). Effects of the Edaravone, a Drug Approved for the Treatment of Amyotrophic Lateral Sclerosis, on Mitochondrial Function and Neuroprotection. Antioxidants.

[157] Yoshida H., Yanai H., Namiki Y., Fukatsu-Sasaki K., Furutani N., Tada N. (2006). Neuroprotective effects of edaravone: A novel free radical scavenger in cerebrovascular injury. CNS Drug Rev..

[158] Fujisawa A., Yamamoto Y. (2016). Edaravone, a potent free radical scavenger, reacts with peroxynitrite to produce predominantly 4-NO-edaravone. Redox Rep..

[159] Dupré-Crochet S., Erard M., Nüβe O. (2013). ROS production in phagocytes: Why, when, and where?. J. Leukoc. Biol..

[160] Riva D.A., de Molina M.C., Rocchetta I., Gerhardt E., Coulombié F.C., Mersich S.E. (2006). Oxidative stress in vero cells infected with vesicular stomatitis virus. Intervirology.

[161] Sada K., Takano T., Yanagi S., Yamamura H. (2001). Structure and function of Syk protein-tyrosine kinase. J. Biochem..

[162] Wu S.Y., Huang J.H., Chen W.Y., Chan Y.C., Lin C.H., Chen Y.C., Liu F.T., Wu-Hsieh B.A. (2017). Cell Intrinsic Galectin-3 Attenuates Neutrophil ROS-Dependent Killing of Candida by Modulating CR3 Downstream Syk Activation. Front. Immunol..

[163] Moens U., Kostenko S., Sveinbjørnsson B. (2013). The Role of Mitogen-Activated Protein Kinase-Activated Protein Kinases (MAPKAPKs) in Inflammation. Genes.

[164] Cooper N., Ghanima W., Hill Q.A., Nicolson P.L., Markovtsov V., Kessler C. (2023). Recent advances in understanding spleen tyrosine kinase (SYK) in human biology and disease, with a focus on fostamatinib. Platelets.

[165] Li Y., Liu J., Sun Y., Hu Y., Cong C., Chen Y., Fang Y. (2025). Targeting p38 MAPK signaling pathway and neutrophil extracellular traps: An important anti-inflammatory mechanism of Huangqin Qingre Chubi Capsule in rheumatoid arthritis. Int. Immunopharmacol..

[166] Jones L.P., Bergeron H.C., Martin D.E., Murray J., Sancilio F.D., Tripp R.A. (2024). Probenecid Inhibits Extracellular Signal-Regulated Kinase and c-Jun N-Terminal Kinase Mitogen-Activated Protein Kinase Pathways in Regulating Respiratory Syncytial Virus Response. Int. J. Mol. Sci..

[167] Borges L., Pithon-Curi T.C., Curi R., Hatanaka E. (2020). COVID-19 and Neutrophils: The Relationship between Hyperinflammation and Neutrophil Extracellular Traps. Mediat. Inflamm..

[168] Ackermann J.A., Nys J., Schweighoffer E., McCleary S., Smithers N., Tybulewicz V.L. (2015). Syk tyrosine kinase is critical for B cell antibody responses and memory B cell survival. J. Immunol..

[169] Marko A.J., Miller R.A., Kelman A., Frauwirth K.A. (2010). Induction of glucose metabolism in stimulated T lymphocytes is regulated by mitogen-activated protein kinase signaling. PLoS ONE.

[170] Vendel A.C., Calemine-Fenaux J., Izrael-Tomasevic A., Chauhan V., Arnott D., Eaton D.L. (2009). B and T lymphocyte attenuator regulates B cell receptor signaling by targeting Syk and BLNK. J. Immunol..

[171] da Cunha A.A., Nuñez N.K., de Souza R.G., Moraes Vargas M.H., Silveira J.S., Antunes G.L., Durante Lda S., Porto B.N., Marczak E.S., Jones M.H. (2016). Recombinant human deoxyribonuclease therapy improves airway resistance and reduces DNA extracellular traps in a murine acute asthma model. Exp. Lung Res..

[172] Tsokos G.C., Lo M.S., Costa Reis P., Sullivan K.E. (2016). New insights into the immunopathogenesis of systemic lupus erythematosus. Nat. Rev. Rheumatol..

[173] Tang R., Yin J., Qin Z., Zhang M., Jia X. (2025). NETs: A new target for autoimmune disease. Front. Immunol..

[174] Chen X.Q., Tu L., Tang Q., Zou J.S., Yun X., Qin Y.H. (2023). DNase I targeted degradation of neutrophil extracellular traps to reduce the damage on IgAV rat. PLoS ONE.

[175] Shak S., Capon D.J., Hellmiss R., Marsters S.A., Baker C.L. (1990). Recombinant human DNase I reduces the viscosity of cystic fibrosis sputum. Proc. Natl. Acad. Sci. USA.

[176] Roesch E.A., Rahmaoui A., Lazarus R.A., Konstan M.W. (2024). The continuing need for dornase alfa for extracellular airway DNA hydrolysis in the era of CFTR modulators. Expert Rev. Respir. Med..

[177] Toma A., Darwish C., Taylor M., Harlacher J., Darwish R. (2021). The Use of Dornase Alfa in the Management of COVID-19-Associated Adult Respiratory Distress Syndrome. Crit. Care Res. Pract..

[178] Holliday Z.M., Earhart A.P., Alnijoumi M.M., Krvavac A., Allen L.H., Schrum A.G. (2021). Non-Randomized Trial of Dornase Alfa for Acute Respiratory Distress Syndrome Secondary to COVID-19. Front. Immunol..

[179] Davis J.C., Manzi S., Yarboro C., Rairie J., McInnes I., Averthelyi D., Sinicropi D., Hale V.G., Balow J., Austin H. (1999). Recombinant human Dnase I (rhDNase) in patients with lupus nephritis. Lupus.

[180] Yong J., Abrams S.T., Wang G., Toh C.H. (2023). Cell-free histones and the cell-based model of coagulation. J. Thromb. Haemost..

[181] Strasser D., Neumann K., Bergmann H., Marakalala M.J., Guler R., Rojowska A., Hopfner K.P., Brombacher F., Urlaub H., Baier G. (2012). Syk kinase-coupled C-type lectin receptors engage protein kinase C-δ to elicit Card9 adaptor-mediated innate immunity. Immunity.

[182] Yan Q., Li K., Chen L., Wang A., Xi Y., Xiao H., Yuan L. (2025). Metabolic reprogramming in efferocytosis. Front. Cell Dev. Biol..

[183] Yu S., Ge H., Li S., Qiu H.J. (2022). Modulation of Macrophage Polarization by Viruses: Turning Off/On Host Antiviral Responses. Front. Microbiol..

[184] Smyth T.R., Brocke S., Kim Y.H., Christianson C., Kovalcik K.D., Pancras J.P., Hays M.D., Wu W., An Z., Jaspers I. (2025). Human Monocyte-Derived Macrophages Demonstrate Distinct Responses to Ambient Particulate Matter in a Polarization State- and Particle Seasonality-Specific Manner. Chem. Res. Toxicol..

[185] Li F., Piattini F., Pohlmeier L., Feng Q., Rehrauer H., Kopf M. (2022). Monocyte-derived alveolar macrophages autonomously determine severe outcome of respiratory viral infection. Sci. Immunol..

[186] Valente M., Dentoni M., Bellizzi F., Kuris F., Gigli G.L. (2022). Specialized Pro-Resolving Mediators in Neuroinflammation: Overview of Studies and Perspectives of Clinical Applications. Molecules.

[187] Pohar J., Lainšček D., Ivičak-Kocjan K., Cajnko M.M., Jerala R., Benčina M. (2017). Short single-stranded DNA degradation products augment the activation of Toll-like receptor 9. Nat. Commun..

[188] Kvietys P.R., Fakhoury H.M.A., Kadan S., Yaqinuddin A., Al-Mutairy E., Al-Kattan K. (2021). COVID-19: Lung-Centric Immunothrombosis. Front. Cell. Infect. Microbiol..

[189] Lax S.F., Skok K., Zechner P., Kessler H.H., Kaufmann N., Koelblinger C., Vander K., Bargfrieder U., Trauner M. (2020). Pulmonary Arterial Thrombosis in COVID-19 with Fatal Outcome: Results from a Prospective, Single-Center, Clinicopathologic Case Series. Ann. Intern. Med..

[190] Ackermann M., Verleden S.E., Kuehnel M., Haverich A., Welte T., Laenger F., Vanstapel A., Werlein C., Stark H., Tzankov A. (2020). Pulmonary Vascular Endothelialitis, Thrombosis, and Angiogenesis in COVID-19. N. Engl. J. Med..

[191] Jing H., Chen X., Zhang S., Liu H., Zhang C., Du J., Li Y., Wu X., Li M., Xiang M. (2021). Neutrophil extracellular traps (NETs): The role of inflammation and coagulation in COVID-19. Am. J. Transl. Res..

[192] Zhu Y., Chen X., Liu X. (2022). NETosis and Neutrophil Extracellular Traps in COVID-19: Immunothrombosis and Beyond. Front. Immunol..

[193] Porter J.C., Inshaw J., Solis V.J., Denneny E., Evans R., Temkin M.I., De Vasconcelos N., Aramburu I.V., Hoving D., Basire D. (2024). Anti-inflammatory therapy with nebulized dornase alfa for severe COVID-19 pneumonia: A randomized unblinded trial. eLife.

[194] Gregoire C., Di Meglio L., Le Cossec C., Ho-Tin-Noé B., Nomenjanahary M.S., Guillaume J., Hamdani M., Losser M.R., Lambiotte F., Le Tacon S. (2025). Multicenter randomized trial assessing efficacy and safety of aerosolized dornase Alfa in COVID-19 ARDS. Sci. Rep..

[195] Åkesson P., Mellhammar L., Rasmussen M., Inghammar M., Jesperson S., Månsson F., Economou Lundeberg E., Walles J., Wallberg M., Frigyesi A. (2025). Aerosolized Dornase Alfa (DNase I) for the Treatment of Severe Respiratory Failure in COVID-19: A Randomized Controlled Trial. Open Forum Infect. Dis..

[196] Ntinopoulou M., Konstantinidis T., Chalkidou A., Papagianni E., Skeva A., Panopoulou M., Chrysanthopoulou A. (2025). IL-1b-Bearing NETs: Bridging Inflammation to Early Cirrhosis in Hepatitis B. Int. J. Mol. Sci..

[197] Li X., Gao Q., Wu W., Hai S., Hu J., You J., Huang D., Wang H., Wu D., Han M. (2022). FGL2-MCOLN3-Autophagy Axis-Triggered Neutrophil Extracellular Traps Exacerbate Liver Injury in Fulminant Viral Hepatitis. Cell. Mol. Gastroenterol. Hepatol..

[198] European Association for the Study of the Liver (2025). EASL Clinical Practice Guidelines on the management of hepatitis B virus infection. J. Hepatol..

[199] Pol S., Lampertico P. (2012). First-line treatment of chronic hepatitis B with entecavir or tenofovir in ‘real-life’ settings: From clinical trials to clinical practice. J. Viral Hepat..

